# How do we describe other people from voices and faces?

**DOI:** 10.1016/j.cognition.2022.105253

**Published:** 2022-10-07

**Authors:** Nadine Lavan

**Affiliations:** Department of Biological and Experimental Psychology, School of Biological and Behavioural Sciences, Queen Mary University of London, Mile End Road, London E1 4NS, United Kingdom

**Keywords:** Person perception, Free descriptions, Face, Voice, Identity discrimination

## Abstract

When seeing someone’s face or hearing their voice, perceivers routinely infer information about a person’s age, sex and social traits. While many experiments have explored how individual person characteristics are perceived in isolation, less is known about which person characteristics are described spontaneously from voices and faces and how descriptions may differ across modalities. In Experiment 1, participants provided free descriptions for voices and faces. These free descriptions followed similar patterns for voices and faces – and for individual identities: Participants spontaneously referred to a wide range of descriptors. Psychological descriptors, such as character traits, were used most frequently; physical characteristics, such as age and sex, were notable as they were mentioned earlier than other types of descriptors. After finding primarily similarities between modalities when analysing person descriptions across identities, Experiment 2 asked whether free descriptions encode how individual identities *differ*. For this purpose, the measures derived from the free descriptions were linked to voice/face discrimination judgements that are known to describe differences in perceptual properties between identity pairs. Significant relationships emerged within and across modalities, showing that free descriptions indeed encode differences between identities – information that is shared with discrimination judgements. This suggests that the two tasks tap into similar, high-level person representations. These findings show that free description data can offer valuable insights into person perception and underline that person perception is a multivariate process during which perceivers rapidly and spontaneously infer many different person characteristics to form a holistic impression of a person.

## 1 Introduction

Perceivers routinely infer information about other people from their voices and faces: Studies have consequently shown that a person’s identity, age, sex, social traits, emotional states, regional origin or ethnicity, among any number of other characteristics, can indeed be perceived with variable degrees of accuracy and reliability from voices and faces alike (Belin, Bestelmeyer, Latinus, & Watson, 2011; Bruce & Young, 1986; Kreiman & Sidtis, 2011; Young, Frühholz, & Schwein-berger, 2020; Yovel & Belin, 2013). Some, mostly early studies of person perception furthermore confirm that perceivers are likely to spontaneously infer a wide range of different person characteristics when describing other people: When asked to select the most fitting characteristics from extensive lists of descriptors (Asch, 1961; Nauts, Langner, Huijsmans, Vonk, & Wigboldus, 2014; Roney & Sorrentino, 1987; Wishner, 1960) or to rate other people’s characteristics based on numerous scales (Addington, 1968; Bayard, Weatherall, Gallois, & Pittam, 2001; Holmgren, 1967; Voiers, 1964; see also Osgood, Suci, & Tannenbaum, 1957), participants will readily select or rate multiple characteristics when prompted to describe a person. This multivariate view of person perception has recently been underlined by neuro-imaging studies of face perception that show that the perception of different person characteristics e.g., information about the age, sex, identity and familiarity of a face can be decoded from the neural response to a single exposure. Notably, there is a time course to the decoding of these characteristics, with age and sex being decodable earlier than information about the identity and familiarity of a face (Dobs, Isik, Pantazis, & Kanwisher, 2019).

Such a multivariate view of person perception from faces and voices is, however, often not reflected in many of the more recent experimental studies on person perception from voices and faces. Most of these studies use experimental designs in which listeners are asked to evaluate or judge a single or a small number of person characteristics of interest. These studies have therefore examined how, for example, the accuracy, reliability, or the time scale associated with forming these different person-related percepts is affected by characteristics of the stimuli, the perceivers, and tasks (e.g., Bruce & Young, 1986; Young and Bruce, 2011; Belin et al., 2011; Burton, 2013; Kreiman & Sidtis, 2011; Lavan, Burton, Scott, & McGettigan, 2019; Mathias & von Kriegstein, 2014). Similarly, studies and reviews have described to what degree the perception of these individual person characteristics may be comparable or may differ across faces and voices (Barsics, 2014; Barsics & Brédart, 2012; Johnson, McGettigan, & Lavan, 2020; Lavan, Mileva, Burton, Young, & McGettigan, 2021; Mileva, Tompkinson, Watt, & Burton, 2020; Stevenage et al., 2013; Stevenage & Neil, 2014; Tatz, Peynircioğlu, & Brent, 2020; Young et al., 2020; Yovel & Belin, 2013). While these findings have been invaluable to our understanding of the perceptual and cognitive mechanisms of person perception from voices and faces, which specific characteristic(s) the perceivers are asked to pay attention to, is usually pre-determined by the experimenter. Whether the particular characteristic of interest is salient or even perceptually-relevant for the description of the specific face and voices presented to participants is unclear. These kinds of studies therefore inevitably guide and potentially restrict the perceivers’ evaluations of other people to the characteristic(s) of interest within a specific experiment. As a result, relatively little is known about how participants might freely describe people based on their faces and voices, especially when looking across visual and auditory modalities: The current study thus sets out to characterise how people are described based on their voices and faces and, crucially, highlight any similarities and differences in person descriptions between the two modalities, while adopting a multivariate approach to studying person perception.

### 1.1 Previous studies using free descriptions of other people

This is, however, not to say that there are no examples of studies looking at person description without clear experimental constraints based on either brief recordings of voices or images of faces: For example, unguided, free descriptions of faces have been collected in previous studies. Most of these studies were primarily interested in understanding trait evaluations from faces, i.e., how viewers form an impression that a person may be assertive, aggressive, or trustworthy. For example, Oosterhof and Todorov (2008) presented participants with images of faces and asked them to “write everything that comes to mind about this person”. The free description data are not the main focus of the experiment and are only used to identify the most frequently used trait descriptors, such that the full dataset is not analysed or described in detail. The authors, however, note that 68% of all descriptors could be summed up under only fourteen trait categories (such as “attractive”, “unhappy”, “sociable”). The remaining descriptions covered physical or appearance characteristics (e.g., sex, age, descriptions of facial features), emotional states, and references to a person’s occupation, attitudes, or preferences. Recently, Lin, Keles, and Adolphs (2021) took a similar approach to Oosterhof and Todorov (2008) and collected free descriptions (based on single words or short phrases) of faces. These free descriptions are also not the main focus of Lin et al.’s study and are thus not formally analysed. From a visualisation of the free descriptions, however, it appears that a wide range of terms was used by participants. Most descriptors appear to be trait evaluations (friendly, caring, kind, confident), although physical properties (male/female, young, pretty, white/black) are also frequently mentioned. Finally, some words relating to the social role and or standing can be seen (e.g., rich, leader).

Some studies have analysed and quantified free descriptions of faces in more detail: Sutherland et al. (2018) collected free descriptions of faces and Chinese and British participants. The authors conduct an analysis of the distributions of descriptors, assigning descriptors to 5 categories: Traits, appearance, age, sex and emotion. The authors report that for British and Chinese participants, ~30% of all descriptors were trait characteristics (such as “friendly”, “capable”, “warm”). This was followed by descriptions of a person’s appearance, age and sex, which each accounted for 8%–20% of descriptors for both participant groups. Collova, Sutherland, and Rhodes (2019) conducted a similar study for descriptions of the faces of children by adults, also reporting trait impressions to be the most frequent descriptors (28% of descriptors), followed by descriptions of appearance (20%) and emotion (15%). Interestingly and in contrast to adult faces, age and sex descriptions were relatively less frequent (7% and 5%, respectively). Similarly, Sutherland, Young, Mootz, and Oldmeadow (2015) collected free descriptions of gender-stereotypical vs counter-stereotypical faces and conducted a valence-focussed content analysis on these free descriptors. The authors here report more negative evaluations for counter-stereotypical faces, with visualisations in the paper again indicating a prevalence of trait descriptions.

For voices, there are very few studies on free descriptions of people from their voices (as opposed to descriptions based on multiple rating scales, see e.g., Holmgren, 1967, Voiers, 1964): One early study by Pear (1931) collected free descriptions of voices based on recordings of 9 voices that were played on national radio in the UK over the course of three days. Listeners then completed a brief questionnaire where they specified the age, sex, profession or occupation, and locality of birth for each of the 9 voices. Listeners furthermore reported whether they thought the voice belonged to a person that is accustomed to leading others. Most interestingly for the purpose of this literature review, listeners were furthermore invited to provide additional freehand comments. As a result, over 600 listeners submitted such additional descriptions. These descriptions were fully reproduced in Pear (1931) but were not analysed in the original paper. An analysis of this rich resource has been, however, recently reported in a blog post (The-SoundBlog, 2019). This blog post notes that a person’s sex was the most commonly used category of descriptions for each of the voices, with descriptions of a person’s dominance being mentioned in 25% of descriptions. Around 30% of descriptions made reference to the occupation of a person, while social class, likability and accent also featured in around 10% of responses for each of these characteristics. Notably, however, these data need to be treated with some caution, as some features of the descriptions are likely to partially reflect the prompts from the questionnaire provided by Pear, e.g., asking to assess whether a person is a leader and asking listeners to comment of a person’s occupation. The data thus do not reflect free description data in a strict sense.

One of the most complete attempts to quantitatively analyse free descriptions of other people can be found in Fiske and Cox (1979). The main aim of the study was to propose a (generalisable) taxonomy through which descriptions of people could be classified. The authors asked participants to give a coherent, written free description of e.g., a stranger they had met on a train, over the course of 10 min. These descriptions were then hand-scored for the types of characteristics the participants used. Based on these scored free descriptions and building on an existing taxonomy derived from children’s descriptions of objects, Fiske and Cox (1979) then derived a detailed taxonomy covering the following categories: 1) Descriptions of a person’s physical appearance (face, body, voice, physical characteristics, such as age and sex), 2) Behaviours (non-verbal communication, speech, occupation, hobbies), 3) Perceived relationships (perceived social network), 4) Social and regional origins (nationality, ethnicity, class, education, occupation), 5) Properties (perceived traits, attitudes, interests) and finally, 6) Context (“where one finds this person”). Using these categories, Fiske and Cox (1979) find that people most frequently use physical appearance descriptors to describe strangers, followed by descriptions of properties, that is personality traits and attitudes. In terms of the temporal ordering of different kinds of descriptions, the data show that physical (appearance) characteristics were often described earlier, while other types of characteristics did not show such a clear temporal trend.

Even in light of the studies reviewed above, it is clear that only relatively little is known about how we describe other people based on their faces, while almost nothing appears to be known about how we spontaneously describe people from their voices. As a result, there is also no data that investigates the degree of similarity of descriptive strategies across modalities. The brief review of the literature above shows that this may, on the one hand, be due to most traditional experimental studies focussing on a single or a small number of characteristics, thus probing identity, age or trait perception separately (often also within a specific modality) as opposed to looking at holistic or multivariate person perception from voices and faces and beyond. On the other hand, the few studies that have asked participants to describe other people freely have tended not to analyse these descriptions quantitatively in detail. Similarly, free description data have to date not been empirically contextualised within other types of data resulting from more commonly used experimental task designs (e.g., forced-choice responses, ratings). Thus, free description data has been somewhat overlooked in person perception research, possibly due to concerns that they may be less readily quantified or interpreted. This, therefore, results in a fundamental gap in our knowledge and in our methodological inventory, where a detailed and quantitative characterisation of how people are described based on their faces and voices, within and particularly across modalities and with only minimal experimental constraints, is largely missing.

### 1.2 Overview of the current study

The current study therefore asks how participants describe a person by collecting free descriptions of a set of 6 female voices and 6 female faces. Experiment 1 examines how different categories of person characteristics (e.g., physical, psychological, social, and stimulus-based vocal or facial characteristics) are used by participants to describe the people behind the faces and voices (see Fiske & Cox, 1979). At this category-level analysis, striking similarities emerged in the current analysis in terms of how 1) faces vs voices and 2) even different identities (within modality) are described. Experiment 2 then examines whether individual descriptors could reveal (perceptual) differences in the characteristics of individual voice and face identities. In light of free descriptor data being not easily quantified or interpreted, Experiment 2 at the same aims to better contextualise findings from the free descriptor data of Experiment 1 within the broader person perception literature by evaluating them in light of more commonly-used experimental tasks in Experiment 2. To do this, measures derived from the most frequent descriptors used for the individual faces and voices (frequency of descriptors, timepoint when descriptors were listed) were linked to perceptual judgements of these characteristics collected in a discrimination task using the same set of faces and voices from Experiment 1. Looking again within and across modalities, Experiment 2 therefore examines whether the data from free descriptions and experimental discrimination tasks encode shared person-related information, which would suggest that both types of data tap into similar representations of person characteristics.

## 2 Experiment 1

This experiment explores which categories of descriptors participants spontaneously use to describe people based on their voices and faces. This question was tackled by collecting free descriptions of sets of voices and faces based on two minutes of exposure to each identity. The free descriptors were then classified into a number of categories to aid the interpretability of the data. The description data were quantitatively analysed within and across modalities in relation to these categories, in terms of the frequency with which different category descriptors were listed as well as the relative position or time course of usage within the exposure period (see also e.g., Fiske & Cox, 1979). Through these data, similarities and differences in how people are described in the two modalities could then be outlined. Based on previous reports of similarities between voice and face perception (e.g., Young et al., 2020; Yovel & Belin, 2013), as well as reports likening face descriptions to descriptions of objects more generally (Fiske & Cox, 1979) broad similarities in the types, frequencies, and time course of words used to describe faces and voices were expected. Based on the previous literature descriptors relating to traits and similar psychological characteristics, I expected these kinds of characteristics to be the most frequently listed category for voices and faces alike.

### 2.1 Methods

#### 2.1.1 Participants

Participants were recruited via the online recruitment platform Prolific.co. All participants were native speakers of English who were born in the UK, spent most of the first 18 years of their life in the UK, and were current residents in England. They were all aged between 18 and 65 years, had no self-reported hearing difficulties (for voice descriptions), correct-to-normal vision (for face descriptions), and an approval rate of over 90% on Prolific.co. The study was approved by the local research ethics committee.

For the descriptions of voices, in total 302 participants were recruited, with participants being randomly assigned to rate 1 of the 6 different voices included in this study (see Materials). From this sample, some participants were excluded: For 5 participants, no description data was recorded due to technical errors or a misunderstanding of the instructions by participants. Three further participants did not follow the instructions and instead of describing the characteristics of the person based on their voice, listed keywords from the sentences read out in the stimulus materials. Beyond this, no exclusions were made. This leaves a final sample of 294 participants who provided useable data (mean age = 34.0 years, SD = 12.0 years, 184 female, 1 other). As such 49 participants described each of the 6 voices included in this study.

For faces, in total 328 participants were recruited to rate 1 of the 6 different faces included in this study. From this sample, 4 participants were excluded from the original participant sample because no description data was recorded due to technical errors or a misunderstanding of the instructions by the participant. This left a final sample of 324 participants who provided useable data, with 54 participants describing each of the different six faces included in this study (mean age = 34.9 years, SD = 11.9 years, 215 female).

### 2.2 Materials

#### 2.2.1 Voices

Voice stimuli comprised recordings of read sentences by 6 female talkers from the LUCID corpus (Baker & Hazan, 2011). The talkers were all monolingual, native speakers of Standard Southern British English and were all aged between 18 and 29 years. Around 40 sentences from each talker were concatenated into a single recording, with short silences separating each sentence, to create a single recording per talker of 2 min of relatively continuous speech. The specific number of sentences per talker varied across identities due to, e.g., differences in speech rate. The sentences had neutral linguistic content (“many cells are studied at school”, “the smoothie was flavoured with peach juice”). All sentences were read in a neutral, unemotional tone of voice throughout by all talkers. Read sentences were chosen for this task to overall reduce the within-person variability across stimuli and thus provide listeners with a relatively stable impression of the characteristics of the person (see Lavan, Mileva et al., 2021 for an example of how within-person variability across voice recordings – and images of faces – can affect trait judgements for the same person).

#### 2.2.2 Faces

To broadly match the nature of stimuli used for the descriptions of voices, participants were presented with dynamic videos of 6 identities reading sentences. These were selected from the GRID corpus comprising audiovisual recordings of sentences with neutral content (e. g., “place green at G6 again”; Cooke, Barker, Cunningham, & Shao, 2006). All 6 identities were female, in their 20s or 30s, and were white. The talkers sat directly in front of the camera and looked at the camera while reading the sentences. Their facial expression was neutral throughout. For the current study, all videos were muted to remove information about the voice of the person. As for the voice recordings, around 40 sentence clips were added together into a single track per talker. This resulted in a 2-min video showing each a head and shoulders view of the talker, with little within-person variability between the sentence clips to provide participants with a relatively stable input of person information.

### 2.3 Procedure

#### 2.3.1 Voices

The study was conducted using the online testing platform Qualtrics. Listeners first read an information sheet and then provided informed consent. After this, participants were informed that they would hear a 2-minute long recording of a person reading out sentences. It was the participants’ task to list words or short phrases that describe the person reading the sentences. Participants were instructed to write down as many different descriptors as they could think of within those 2 minutes. They were asked to avoid long descriptions and focus on brief, ideally single-word descriptions. Participants were told that the content of the sentences in the voice recordings was not informative, was thus irrelevant to the task, and should not influence their responses. After reading these instructions, participants then proceeded to complete the task: At the top of the screen, a countdown of 2 minutes was shown, beneath which listeners were asked to start replaying the voice recording via a button click. Below the countdown and the voice recording button, 30 textboxes were provided on the page. Participants were asked to enter their descriptions into these text boxes. No participant provided >19 descriptions within the 2 minutes, suggesting that the 30 boxes were sufficient. Each listener provided free descriptions of 1 of the 6 voices in the study.

After the 2 minutes had passed, participants were automatically forwarded to a new page. Here, listeners were asked to indicate the sex of the voice and indicate the language spoken by the voice as a basic attention check. They were furthermore asked about their strategy to complete the task, whether they felt like they had enough time to describe the voice, and whether they encountered any technical difficulties. These data were not formally analysed but did also not provide any cause for concern: All participants passed the attention checks; no technical difficulties were reported after exclusions (see above) and most participants felt like they had adequate time to describe the voice.

#### 2.3.2 Faces

The procedure for collecting descriptions of faces was identical to the one reported to voices. The only difference was that participants started a silent video showing the identities instead of starting a recording of the voices. The video was placed in the browser window such that it would be visible to participants on a number of screen resolutions throughout while writing down descriptors in the text boxes.

### 2.4 Data cleaning

Data cleaning was conducted for descriptions of voices and faces in the same manner: All incomplete final words were removed (e.g., “int” or “unin”). All descriptors were made lower case, spelling errors were fixed (e.g., “elagent” to “elegant” and “consise” to “concise”), and hyphenation was standardised. Qualifiers (e.g., “fairly”, “quite”, “very”) were removed for the purpose of this study. Descriptors that included more than one characteristic were split (e.g., “middle-aged woman” was split into “middle-aged” and “woman”). If listeners listed the exact same descriptor multiple times, only the first instance was retained. To further standardise responses, phrases such as “kind person” or “intelligent person” were reduced to the adjective only. Phrases describing a feature of a person (e.g., “like a teacher”, “is a teacher”, “could be a teacher”) were, where easily possible, reduced to including the feature only (i.e., “teacher”). Near-synonyms or descriptors that differed in their suffixes (e.g., “bored” vs “boring”) were retained as it was unclear how to best merge them.

### 2.5 Categorisation of descriptors

**Fig. 1:**
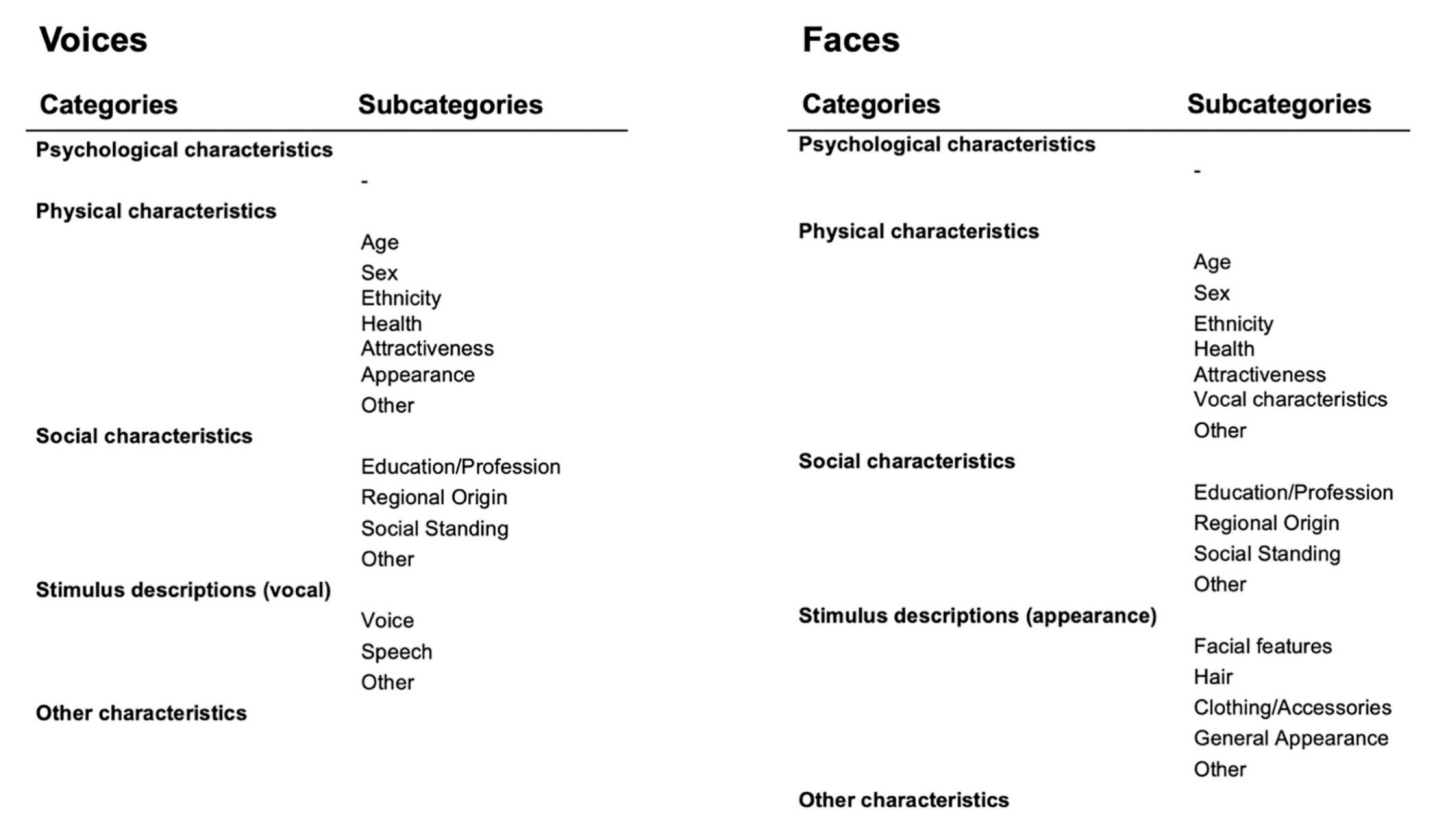
Overview of the categories and subcategories used for free descriptors data for voices and faces.

To make the rich data set of free descriptors of voices and faces more readily interpretable, descriptors were classified into a framework of categories and subcategories that were similar for faces and voices. These categories and subcategories are outlined in Fig. 1 below and were loosely derived from other frameworks detailing on judgements made from voices and faces (Fiske & Cox, 1979; Kreiman & Sidtis, 2011; Oosterhof & Todorov, 2008). Categories include physical characteristics (e.g., descriptions of age, sex, ethnicity, health, and attractiveness), psychological characteristics (e.g., descriptions of moods, emotions, character traits), social characteristics (e.g., descriptions of regional origin, social standing, and a person’s education/profession), and finally, a category including descriptions of features of the stimuli (faces/voices, e.g., descriptions of the speaking style or facial features) themselves. As such, the categories were chosen to cover the most frequent person-related judgements associated with both voices and faces, thus creating a relatively simple framework that allows for the categorisation of most descriptors used for voices and faces alike.

Stimulus descriptions were separated from other categories as stimulus descriptions did not include substantial inference about the person beyond the visual/auditory input, while all other descriptions reflected more inferences being made. That is, “high-pitched” would be classed as a stimulus description, while “female”/“girly”/“happy”, which are all associated with higher pitch in voices, would be classed as physical or psychological characteristics. Similarly, for faces, “wrinkly” would be classed as a stimulus characteristic while “old” or “worried” would be classed as physical or psychological characteristics for faces. While there is substantial overlap for stimulus characteristics and physical characteristics for faces (e.g., “blonde hair”, “long nose”), stimulus characteristics for voices are often less readily classified into the other categories (e.g., “monotone”, “fast speaking”), speaking for a separation of this category.

**Fig. 2:**
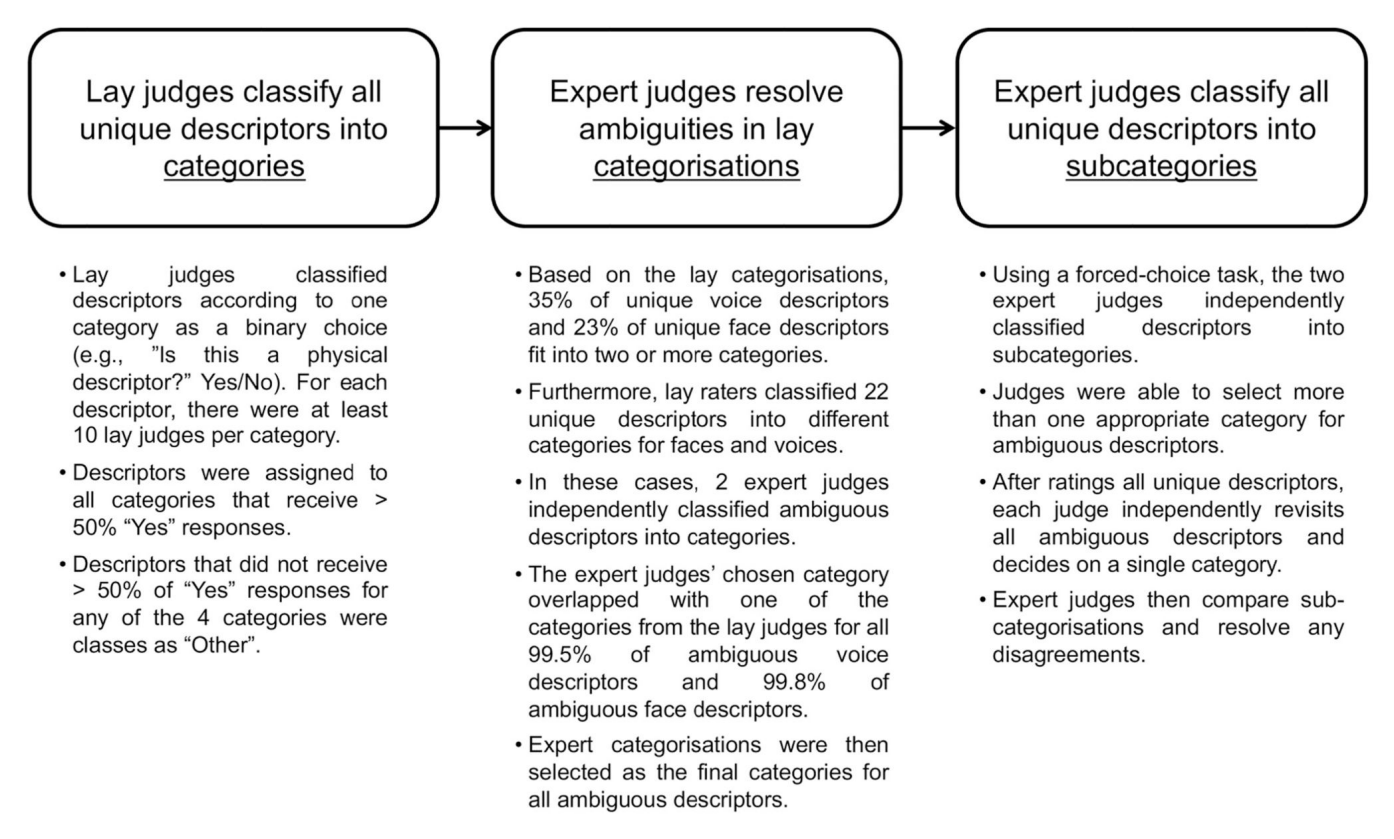
Overview of the classification process for the free descriptors data into categories and subcategories.

As can be seen from the flowchart presented in Fig. 2, the four broad categories were assigned based on the classification by groups of lay judges. Two expert judges (the author of this paper and another expert in voice identity perception) then systematically resolved ambiguities arising in these lay classifications where necessary. Furthermore, the two expert judges subcategorised all descriptors based on the categories assigned by lay judges. A more detailed description of the categorisation process is given in Supplementary Analysis 1.

To be able to compare the categorisation by human judges described above to a computational clustering solution, a hierarchical agglomerative clustering (HAC) analysis based on the semantic similarities of the descriptors was conducted for voices and faces separately. The HAC is presented in more detail in Supplementary Analysis 2. Briefly, the analysis showed that clusters identified via the HAC included similar broad themes (psychological characteristics, stimulus [voice/appearance] characteristics, physical characteristics, and social characteristics) emerge from the computational clustering solution. Notably, however, there were several issues, limiting the interpretability of the HAC in the context of this study: For example, any clusters emerging from the computational solution included a wide range of the descriptors within the same loosely cluster (e.g., “blonde”, “girl”, “researcher” and “London”). Similarly, how individual descriptors fit into clusters differs partially for voices and faces, making direct comparisons across modalities difficult. Finally, the number of descriptors entered into the hierarchical cluster analysis (e.g., entering all descriptors vs only descriptors mentioned >3 times) substantially affected clustering solutions. While such computational clustering approaches will have merit in other contexts, classifications based on human judgements were used for the current manuscript. The HAC presented in the supplementary materials may nonetheless be seen as a way of partially validating the categorisation methods outlined above, as broadly similar themes emerge.

### 2.6 Data analysis

All data were analysed using R. All Linear Mixed Models (LMMs) were run in the R package lme4 (Bates, Mächler, Bolker and Walker, 2014) where possible. Model structures for individual analyses are described in more detail within the Results section. The significance of LMMs was established by comparing a full model, including all fixed effects and interactions of interest, to a reduced model from which an effect of interest had been dropped. Pairwise post-hoc tests for any significant effects arising from the LMMs that included more than two levels were implemented using the R package emmeans (Lenth, 2016). All results were Bonferroni-corrected for multiple comparisons.

## 3 Results

### 3.1 How many descriptors do participants list for voices vs faces?

For voices, participants overall provided 2749 descriptors for the 6 voices, with 723 of them being unique. On average, each participant provided 9.4 descriptors (*SD* = 3.1, *range* = 3–19). Participants on average listed a broadly similar number of descriptors for each of the six voices (mean number of descriptors per voice: 8.1–10.0 descriptors). For faces, a similar picture emerged: Participants overall provided 2976 descriptors for the six faces, with 904 of them being unique. Each participant provided on average 9.2 descriptors (*SD* = 3.2, *range* = 3–24) and, as for voices, participants listed on average a broadly similar number of descriptors for each of the 6 faces (mean number of descriptors per face: 8.8–9.5 descriptors).

LMM predicting the number of descriptors listed by each participant based on modality (voices vs faces) were run to statistically compare the number of descriptors for voices vs faces. In these models, modality was entered as the sole fixed effect. Voice/face identity was entered as a random effect. This LMMs confirmed that the number of descriptors listed by participants did not differ for voices and faces (χ^2^(1) = 0.74, *p* = .389, β = −0.23, CI[−0.79–0.34]).

Furthermore, for faces, a Kruskal-Wallis^1^ test showed that participants listed a similar number of descriptors for each of the different identities (χ^2^(5) = 2.74, *p*. = 740). For voices, there were differences between the different identities (χ^2^(5) = 11.49, *p* = .042). Post-hoc Mann-Whitney tests, however, indicated that all voices were comparable to one another (all U’s > 1534, z < 2.82, ps > 0.004, Bonferroni corrected for 15 comparisons). Even without correction for multiple comparisons, 11 of the 15 comparisons were not significant (all ps > 0.185). The 4 comparisons that were significant (i.e., *p* < .05), all involved one voice (Voice 3), which received fewer descriptors compared to the remaining voices (8.1 descriptors), while the other 5 voices received between 9.4 and 10.0 descriptors. Thus, independent of the modality of the stimulus and the specific identity, participants appear to list a similar number of descriptors for a face or a voice.

### 3.2 Which categories of person characteristics are used to describe other people from voices and faces?

Based on the categorisations, I further examined which person characteristics were used to describe people based on their voices and faces. To do this, the free description data were explored based on the four categories (psychological, physical, social, and stimulus [vocal/appearance] characteristics) for voices and faces respectively, then moving on to subcategories (e.g., age, sex, ethnicity, etc. for physical characteristics) and individual descriptors. In this analysis, I first describe the degree to which participants used descriptors from across the different categories, for voices and faces respectively. This is followed by a description of the similarities and differences between how voices and faces were described.

#### 3.2.1 Categories

##### 3.2.1.1 Voices

**Fig. 3:**
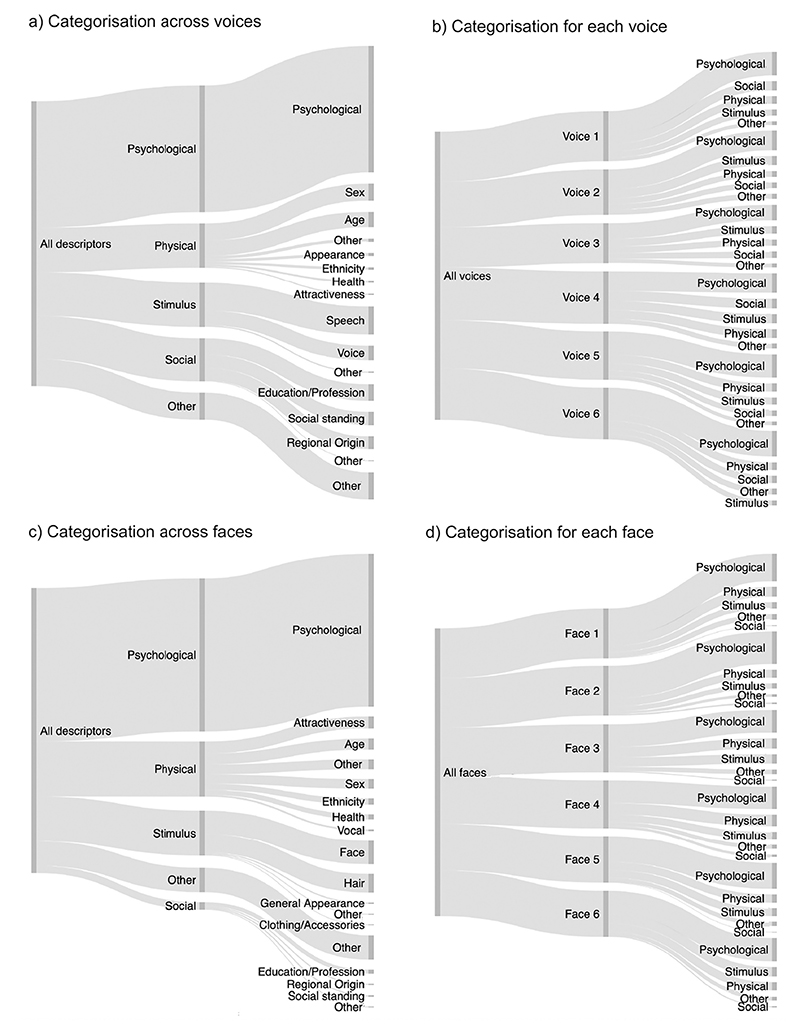
a) + c) Sankey plots visualising how many descriptors fall into the different categories and subcategories for voices and faces across all identities. b) + d) Sankey plots showing how many descriptors are falling into different categories for each individual identity. The thickness of the grey lines is proportional to the number of descriptors listed for a certain (sub)category relative to the other (sub)categories at that level: The wider the line, the more descriptors were used. Sankey plots were created via sankeymatic.com.

Of the 2749 descriptors provided by listeners, 44.3% of descriptors referred to psychological characteristics and 15.5% of descriptors referred to stimulus characteristics. Another 15.5% of descriptors referred to physical characteristics while 15.1% of descriptors referenced social characteristics (Fig. 3a). The remaining descriptors were unclassifiable, thus categorised as “Other” (9.5%, see Methods).

Distributions of descriptors for each of the voices followed a similar pattern (see Fig. 3b): For each of the 6 voices, participants most frequently referred to psychological characteristics, followed by stimulus, physical, and social characteristics. As observed across all voices, the latter 3 categories were all mentioned with relatively similar frequencies as indicated by the similar thickness of the lines associated with the characteristics in Fig. 3b. Percentages per voice are provided in Supplementary Analysis 3. The relative use of the categories of descriptors (psychological, stimulus, physical, and social) therefore does not appear to be voice-specific but may be generalisable – at least within the current set of 6 voices.

##### 3.2.1.2 Faces

The results of the distribution of descriptors across the categories for faces are visualised in Fig. 3c: Of the 2976 descriptors of person characteristics provided by participants, 53.6% of descriptors referred to psychological characteristics and 19.3% of descriptors referred to physical characteristics. Another 15.8% of descriptors referred to the stimulus characteristics, referencing the appearance of the face of a person, while only 2.6% of descriptors were categorised as primarily social characteristics, thus referring to the social standing, regional origin or degree of education of a person.

As for voices, the distributions of how the descriptors fell into the different categories for each individual face were similar to the pattern observed across all faces (see Fig. 3d): Participants most frequently referred to psychological characteristics. This was followed by descriptions of a person’s stimulus and physical characteristics, with there being somewhat more descriptors of stimulus than physical descriptors for all but one of the faces (20.9% stimulus characteristics vs 17.9% physical characteristics for Face 6). The least used category of descriptors for all faces was social characteristics. Percentages per face identity are provided in Supplementary Analysis 3.

##### 3.2.1.3 Faces vs voices

To compare whether there are differences between how frequently the different descriptor categories were used for voices vs faces, the percentage of descriptors per category for each participant was computed. Four LMMs were then run, one for each category of descriptors (psychological, social, physical, stimulus): For these 4 models, the percentage of descriptors used for the relevant category by each participant was predicted from the stimulus modality (faces vs voices). Stimulus modality was thus included as a fixed effect and the specific voice/face identity as a random effect.

These models showed that participants proportionally listed more psychological characteristics for faces than for voices (χ^2^(1) = 753.06, *p* < .001, β = 12.22, CI[5.54–18.91]). Similarly, they listed more physical characteristics for faces than for voices, although this effect is much smaller than the effect for psychological characteristics (χ^2^(1) = 6.10, *p* = .014, β = 3.69, CI[0.89–6.52]). At the same time, participants listed fewer social characteristics for faces than for voices (χ^2^(1) = 26.64, p < .001, β = − 12.85, CI[− 15.61 − – 10.08]). The percentage of descriptors directly referring to stimulus characteristics was similar across modal-ities (χ^2^(1) = 1.53, *p* = .216, β = −2.59, CI[−6.94–1.76]).

##### 3.2.1.4 Subcategories and individual descriptors

Fig. 3a and c show a breakdown of how different subcategories were used for voices and faces respectively. As can be seen from these figures, broad similarities in how descriptors are distributed across the different subcategories across modalities alongside some differences. For example, age and sex descriptors are common descriptors for faces and voices alike for physical descriptors. However, for faces, descriptors referring to the attractiveness of a person (“pretty”, “cute”, “attractive”) are as common as age and sex descriptors, while attractiveness is barely mentioned for voices. Participants also frequently described a person’s perceived social standing or class (“middle-class”, “posh”) based on their voice, these descriptions are largely absent for faces. Similarly, descriptions of regional origins are more prevalent for voices than for faces. For a more detailed descriptive account of these data for voices and faces in the supplementary materials (Supplementary Analysis 4). No statistical analysis of these data is provided because there are often insufficient data points across the subcategories. For the same reason, the more fine-grained descriptive observations should also be treated with caution. Figs. 5 and 6 below provide illustrations of the individual descriptors used across all faces and voices.

### 3.3 Which characteristics are prioritised in time for voices and faces?

In the current experiment, participants were presented with a 2-minlong stimulus of a voice recording or a silent video of a face. During these 2 minutes, participants sequentially listed descriptors of the person they saw or heard. Thus, information about the position or time course of when different descriptors may prove informative (see Fiske & Cox, 1979 for a similar analysis). The next analysis therefore asked whether participants list some categories of descriptors earlier than others and whether this time course information differs across modalities. While to this point the descriptors have only been examined via frequency-related measures, this position or time-course-related measure offers an additional perspective on how participants choose to describe voices and faces: Much like descriptor categories that are listed frequently may be considered to be important during person perception, descriptors that are listed early on could also be viewed as being prioritised during person perception from voices and faces.

### 3.4 Voices

The relative position of the different descriptors listed was calculated for each participant: For this purpose, the descriptors were labelled by position (e.g., Position 0–9 for a participant who has listed 10 descriptors). This position index was then divided by the total number of descriptors listed by this participant - 1, creating a continuous index of relative position that ranges from 0 (first position) to 1 (last position). To test whether different descriptor categories were on average listed earlier or later in relation to one another, a linear mixed model was built, which included the relative position of each descriptor as the dependent, the descriptor category (psychological, physical, stimulus, social) as a fixed effect, and voice identity and participant as random effects with no random slopes. The model showed that descriptor category indeed had a significant effect on when descriptors were listed for voices (χ^2^(4) = 53.33, *p* < .001). Post-hoc tests showed that for voices, physical descriptors were listed earlier than psychological, stimulus, and social descriptors (βs > −0.10, ps < .001; see Fig. 7, left side). Psychological, stimulus, and social descriptors were, however, listed at a similar position within descriptor lists (βs < 0.04, ps > 0.105).

### 3.5 Faces

The same analyses as for voices was run for faces, also building a model with the relative position of each descriptor as the dependent, descriptor category (psychological, physical, stimulus, social) as a fixed effect, and face identity and participant as random effects. For faces, descriptor category also had a significant effect on relative position (χ^2^(4) = 36.41, p < .001). Here, a similar picture to what was observed for voices emerged, where physical descriptors were listed earlier than psychological, stimulus, and social descriptors (βs > − 0.06, ps = 0.004, see Fig. 7, right side). Stimulus, psychological and social descriptors were again listed similarly early (βs > − 0.06, ps > 0.044, an effect that was not significant after correction for 6 comparisons).

### 3.6 Voices vs faces

To test whether voices and faces differ in how early descriptors for the different categories are listed, four additional LMMs were run, one for each category of descriptors shared across the 2 modalities (psychological, social, physical, and stimulus characteristics).

These models showed that the relative position of descriptors was similar for psychological characteristics (χ^2^(1) = 0.02, *p* = .888, β = 0.00, CI[−0.02–0.03]), physical characteristics (χ^2^(1) = 0.94, *p* = .333, β = 0.03, CI[−0.08–0.02]), social characteristics (χ^2^(1) = 1.37, *p* = .242, β = −0.05, CI[−0.14–0.04]), and stimulus characteristics (χ^2^(1) = 3.38, *p* = .066, β = 0.05, CI[−0.01–0.11]).

## 4 Discussion

In Experiment 1, several similarities are apparent between how participants describe other people based on their voices and faces. Participants listed similar numbers of descriptors for each of the individual voices and faces, with the distributions of descriptors across different categories also being similar across modalities – and, descriptively speaking, even across individual identities. Crucially, however, despite these similarities, participants tended to use a wide range of descriptors when prompted to describe people from their voices and faces, tapping into various categories and thus person characteristics. Different categories were then used with different frequencies: Psychological characteristics were the most frequently used category by far for both voices and faces alike. When looking across modalities, participants used proportionally more psychological descriptors for faces than for voices. Stimulus-based descriptions of a person’s facial appearance or voice and physical characteristics were used similarly often in relation to the other categories within modality. Across modalities, there was no difference in how frequently stimulus characteristics were listed for voices and faces, although participants used more physical descriptors for faces than voices. The latter finding may be explained by participants listing many more attractiveness-related descriptions in the visual modality. Social characteristics, as they were defined in the current study, were used similarly frequently to physical and stimulus characteristics for voices but were mentioned least frequently for all face identities. In addition to these findings based on frequency-related measures, physical characteristics, such as the age and sex of a person, were mentioned relatively earlier than the remaining characteristics for voices and faces alike. Thus, while some differences between modalities emerged (e.g., faces elicited fewer social descriptors than voices, there were more psychological descriptors for faces than for voices), the similarities across modalities often outweigh the observed differences.

Our findings for voices and faces generally align with reports from previous studies that have collected free descriptions of other people based on faces or stimulus-free written instructions: For example, Oos-terhof and Todorov (2008) report that traits (i.e., descriptors classed as “psychological characteristics” in the current study) were by far the most common type of descriptors for faces – with the 14 most frequent “trait categories” (e.g., trustworthy, friendly) accounting for 68% of all descriptions. Sutherland et al. (2018) also report that trait descriptions (i.e., psychological characteristics in the current study) are the most commonly used type of descriptors during free description tasks of faces by British and Chinese adults alike, while Collova et al. (2019) report the same prevalence of trait descriptions for faces of children. Similarly, Fiske and Cox (1979) report that psychological characteristics (called “personality” characteristics in their paper) accounted for most descriptors after descriptors of physical characteristics (which include both physical and stimulus-based characteristics in the current study).

Finally, Lin et al. (2021) also find that freely generated descriptions of faces frequently included what is referred to as psychological characteristics in the current study. In line with the findings from Experiment 1, Fiske and Cox (1979) also report a similar findings for the time course or position of physical descriptors (vs all other categories): In both their study and the current study, most physical descriptors are listed early on and drop off steeply afterwards, while for the remaining categories of descriptors, trajectories are relatively flat.

When trying to review the current findings in light of the existing literature, a key difficulty when trying to categorise natural language data for further quantitative analysis becomes apparent. Categorisation frameworks differ from paper to paper with, e.g., age and sex being sometimes viewed as their own categories (Collova et al., 2019; Sutherland et al., 2018), sometimes categorised among social roles (Oosterhof & Todorov, 2008) and sometimes as physical characteristics (the current manuscript, Fiske & Cox, 1979; Kreiman & Sidtis, 2011). Similarly, individual descriptors will only rarely fit clearly into one category without also at least partially fitting into another, with e.g., “feminine” referring to someone’s physical characteristics, while also likely tapping to some degree into psychological or social characteristics (see also the computational cluster analysis reported in the supplementary materials). Thus, even though categorisation frameworks and individual categorisations of descriptors for all studies were validated by multiple judges/raters, these observations show that natural language data cannot be readily and objective categorised and analysed without researchers imposing some assumptions on these rich data. While there are idiosyncrasies of coding frameworks across different studies, it is worth noting that this does however not necessarily detract from the interpretability of the findings. As long as coding frameworks and the underlying data are described in sufficient detail, it is relatively straightforward to directly compare findings despite the differences in coding frameworks. Thus, for example, key findings in the current study, such as “psychological characteristics”/traits being the most frequently used type of descriptors, appear to replicate readily across studies and categorisation frameworks.

So far, the free description data have been examined and quantified based on categories, both in terms of the relative frequency and the relative position (i.e., time point) at which these categories were used within each participant’s list of descriptors. Both types of measures could be interpreted as indexes of how salient or prominent the features captured by different descriptors are during person perception: If this is the case, psychological properties could therefore be viewed as being an important type of characteristic during person perception because psychological descriptors are listed more frequently than any other category (across all positions or timepoints) in participants’ lists of descriptors. This interpretation would align with, and would also validate, the traditional focus of the face and voice perception literatures on trait perception and evaluations. Similarly, however, physical characteristics were shown to be mentioned on average earlier than the other categories (albeit mentioned less frequently than e.g., psychological characteristics overall), thus also potentially marking them out as highly salient characteristics. Both interpretations of these data can be simultaneously valid, with both types of measures potentially tapping into at least partially distinct aspects of how salient or important the different categories of descriptors are during person perception. To empirically confirm the validity of these interpretations, more data is, however, necessary.

From the findings in Experiment 1, thus several questions arise or remain unanswered. First, the free descriptor data have up to this point only been examined in relation to categories and across different identities. These analyses have primarily revealed similarities in how voices and faces generally are described by participants. Within the free descriptions in this study, we can, however, also examine how individual descriptors are used across the different identities: The individual descriptors listed for each of the identities should, for example, hold information about the prominent characteristics of this specific identity, thus providing an opportunity to explore differences between different identities. Second, From a methodological and theoretical point of view, the insights already gained from the free description data are certainly valuable in themselves. They are, however, somewhat difficult to evaluate – both in isolation and in light of existing experimental work. For example, we do not yet know whether descriptor frequency and descriptor position are indeed meaningful indexes of the relative prominence or salience of different person characteristics (see above). Similarly, we do not know whether the free descriptions tap into similar processes or representations as some of the more commonly used experimental person perception tasks do. Experiment 2 attempts to shed additional light on these questions.

## 5 Experiment 2

Experiment 2 tried to address whether comparing how individual descriptors are used across different identities may additionally reveal how these individual voices and faces differ from one another. Additionally, this experiment examined how free descriptor data may relate to person perception judgements collected via other experimental tasks. To do this, the free descriptors were linked to data from voice/face discrimination tasks that are known to track perceptual differences between pairs of voices and/or faces. Through this process, potentially shared information encoded in these two different types of data could be identified. Experiment 2 furthermore examined whether e.g., frequency-or position-related measures derived from the free description do indeed tap into concepts such as the prominence of characteristics in a person’s voice or face.

Six of the most frequently listed descriptors for voices and faces respectively were selected, 3 of which were shared across modalities, while the remaining three were modality-specific. A series of within-modality voice and face discrimination tasks on each of these descriptors was run, alongside three crossmodal face-voice discrimination tasks on the descriptors shared across faces and voices.

In the discrimination tasks, participants were asked to indicate which of two voices, two faces or the face and voice in a pair sounded or looked e.g., younger, more female, or more bored. Based on the existing free descriptor data from Experiment 1, the 1) total frequency and 2) mean position of each descriptor within the list of descriptors were calculated for each voice and face. To link these two types of measures to the discrimination judgements, the pairwise differences in each descriptor frequency and position were then calculated, for all possible identity pairings. These pairwise differences in descriptor parameters were then linked to the corresponding pairwise binary judgements from the discrimination tasks.

If free descriptions of people indeed hold information about how individual identities differ from one another and there is furthermore shared information between free descriptions and discrimination judgements, there should be significant relationships between the measures derived from free descriptor data and the discrimination tasks. This study was not preregistered.

### 5.1 Methods

#### 5.1.1 Participants

As in Experiment 1, participants were recruited via the online recruitment platform Prolific (Prolific.co). All participants were native speakers of English who were born in the UK, spent most of the first 18 years of their life in the UK and were current residents in England. They were all aged between 18 and 40 years and had an approval rate of over 90% on Prolific. For the voice discrimination, participants furthermore reported not having hearing difficulties, while for the face discrimination tasks, participants reported having normal or corrected-to-normal vision. The study was approved by the local research ethics committee.

For the voice discrimination tasks, the data of 144 participants (mean age = 28.1 years, SD = 5.9 years; 91 female) were analysed. Before arriving at this sample, one participant was excluded from the original participant sample of 145 participants as they failed >20% of the vigilance trials (see Methods). For the face discrimination task, the data of 162 participants (mean age 26.9 years, SD = 6.6 years; 119 female) were analysed. Before arriving at this sample for faces, 16 participants were excluded who failed the vigilance trials (see Methods) from the original sample of 178 participants. For the crossmodal discrimination task, the data of 91 participants (mean age 31.9 years, SD = 10.1 years; 69 female) were analysed. There were no exclusions based on vigilance trials (see below).

### 5.2 Materials

#### 5.2.1 Voices

Two read sentences were selected for each of the 6 voices from the recordings that were used in Experiment 1. With there being relatively little within-person variability in the recordings of the voices, these 2 sentences were thus considered to be representative of the voice of each identity.

#### 5.2.2 Faces

As for the voices, 2 representative videos were selected for each of the 6 faces from Experiment 1.

#### 5.2.3 Procedure

##### 5.2.3.1 Voice discrimination

The study was implemented on the online testing platform Gorilla.sc (Anwyl-Irvine, Massonnié, Flitton, Kirkham, & Evershed, 2020). After reading the information sheet and providing informed consent, participants were asked to adjust the volume of their sound output to a comfortable level. After this, participants were randomly assigned to one of six discrimination tasks: In each discrimination task, participants were asked to judge a pair of voices in relation to 1 of 6 descriptors taken from the free descriptions: 1) Age (“Which of the voices sounds *younger*?”, 2) sex (“Which of the voices sounds more *female*?”), 3, boredom (“Which voice sounds *more bored*?”), 4) social status (“Which voice sounds *posher*?”), 5) level of education (“Which voice sounds more *educated*?”), 6) calmness (“Which voice sound *calmer*?”). These 6 descriptors were chosen from the 10 most frequently used descriptors for voices and were selected to span a range of categories and subcategories, such that they include physical, psychological, and social characteristics.

In each of these discrimination tasks, participants were presented with a pair of voices and registered their response (“Voice 1”, “Voice 2”) via a click on a response button shown on the screen. The pairs of voices presented to participants per trial included exhaustive pairings of the 6 identities, excluding pairings that included the same identity twice. Each identity was furthermore represented twice by the 2 representative recordings (15 identity pairs x 2 sentences). The order of voices in each pair was additionally counterbalanced: As such, each pair of recordings was repeated twice, such that both possible orders of recordings were sampled. Participants overall completed 60 experimental trials each. There were additionally 6 catch trials, where instead of one of the female voices, a computer-generated male voice instructed listeners to respond with either “Voice 1” or “Voice 2”. Participants had been briefed to follow these verbal instructions.

##### 5.2.3.2 Face discrimination

The procedure for the face tasks was identical to the one reported for voices with the voice stimuli being replaced by face stimuli and the catch trial being changed from verbal instructions to select a specific response option to visual, written instructions. Furthermore, the descriptors being assessed by participants were based on the 10 most frequently listed descriptors for faces from Experiment 1. Three of the descriptors used for faces overlapped with the ones selected for voices (1) age, 2) sex, 3) boredom) and the remaining three descriptors were unique to faces: 4) tiredness (“*Which person looks more tired?”*), 5) ethnicity *(“Which person looks whiter?”*) and 6) hair colour *(“Which person looks more blonde?”*).

##### 5.2.3.3 Crossmodal discrimination

The procedure for the crossmodal discrimination task was almost identical to the one reported for voices and faces. The only differences were that during each discrimination trial, the pair of stimuli that was presented to participants included one muted video of a face and one recording of a voice (as opposed to 2 faces or 2 voices). Exhaustive pairings of the face and voice identities across modalities within the pair resulted in 36 experimental trials. Each identity was presented twice. The order of modalities was counter-balanced across the two presentations. There were furthermore 8 catch trials, 4 visual vigilance trials (see *Face discrimination*) and 4 auditory vigilance trials (see *Voice discrimination*) to ensure that participants were paying attention to both modalities. In total, there were therefore 80 trials per task. In this crossmodal discrimination task, judgements were collected for the three of the descriptors that were present in the 10 most frequent descriptors for both faces and voices alike: 1) age, 2) sex, and 3) boredom.

### 5.3 Data processing and analysis

Data for each of the descriptors were analysed using separate generalised linear mixed models (GLMM) in the R package lme4 (Bates, Mächler, Bolker, & Walker, 2014). These models tested whether the absolute difference in how many times each of the 6 descriptors of interest (“young”, “female”, “bored”, “posh”, “educated”, “calm” for voices, “young”, “female”, “bored”, “blonde”, “white”, “tired” for faces, (“young”, “female”, “bored” for the crossmodal task) were mentioned for each voice and/or face could predict the binary judgements from the discrimination task. For the binary judgements from the discrimination task, the identity in a pair for whom the descriptor frequency was higher was always coded as 0 and the identity that had the lower descriptor frequency was always coded as 1.

As in Experiment 1, Experiment 2 additionally assessed whether relative descriptor position could predict participant responses in the face and voice discrimination tasks. As for the models using descriptor frequency as a predictor, the identity in a pair for whom the descriptor position was higher (i.e., the descriptor was listed relatively later) was coded as 0 and the identity that had the lower relative position (i.e., the descriptor was listed relatively earlier) was coded as 1. For the descriptor “blonde” for faces, 2 of the 6 identities were never labelled as blonde. Therefore, no data for descriptor position was available for these identities and all pairs including these two identities were excluded from that model.

In practice, the coding systems applied to the responses should be interpreted in the following manner in relation to descriptor frequency: If in each pair, participants clearly perceived the identity that had received the descriptor of e.g., “young” more frequently as being the younger identity in the pair, responses would on average trend towards 0. If participants could not reliably perceive a difference in the characteristic in question for the two identities in a pair and would thus make random responses, responses would on average trend towards 0.5. If participants would consistently perceive the identity with the lower descriptor frequency than e.g., younger, responses would on average trend towards 1. If there is a relationship between descriptor frequency and discrimination judgements, where the two types of data share information about the voice or face, a negative relationship should be apparent for these analyses. These differences in descriptor frequencies and discrimination responses are visualised in Fig. 7, for voices and faces respectively. Ranges of the descriptor frequencies are provided in Supplementary Table 4.

For the measure derived from the descriptor position, the opposite direction of effects should emerge: If participants reliably perceived the identity for which the descriptor of e.g., “young” was listed earlier in time as being the younger identity in the pair, responses would on average trend towards 0. If participants would consistently perceive the identity for whom “young” was listed later in time as “younger”, responses would on average trend towards 1. If there is a relationship between descriptor position and discrimination judgements, where the two types of data share information about the voice or face, a positive relationship should be apparent. These differences in descriptor position and discrimination responses are visualised in Fig. 8 for voices and faces respectively.

Our statistical models tested whether descriptor frequency or descriptor position could predict participants’ discrimination task responses, separately for each descriptor investigated here and within the two modalities:

Coded discrimination response ~ absolute difference in descriptor frequency + (1|participant)

Coded discrimination response ~ absolute difference in descriptor position + (1|participant)

The models run for crossmodal judgements were identical when predicting discrimination responses from the differences in descriptor position. For models predicting discrimination responses from the descriptors frequency, the absolute difference in relative descriptor frequencies was used since the total number of descriptors differed for faces and voices due to slightly different participant numbers.

As in Experiment 1, statistical significance was established via log-likelihood tests between the full and a null model. A null model was created by dropping the fixed effect of descriptor frequency or descriptor position from the full model. Note that the coding systems for the dependent variable make it impossible to include both differences in descriptor frequency and differences in descriptor position within the same model.

## 6 Results

### 6.1 Can differences in descriptor frequency describe how identities differ from one another?

#### 6.1.1 Voices

The GLMMs showed that for 4 of the 6 descriptors of voices (“young”, “female”, “bored”, and “educated”), differences in descriptor frequencies could indeed significantly predict participants’ discrimination responses, as indicated by the negative relationship plotted in Fig. 8a (bottom row).

Specifically, there were negative relationships between descriptor frequencies and discrimination judgements of age (“young”: χ^2^(1) = 125.66, *p* < .001, OR = 0.90, CIs[0.88–0.92]), sex (“female”: χ^2^(1) = 164.55, p < .001, OR = 0.76, CIs[0.73–0.80]), degree of education (“educated”: χ^2^(1) = 40.39, *p* < .001, OR = 0.90, CIs[0.87-0.93]), and degree of boredom (“bored”: χ^2^(1) = 349.61, *p* < .001, OR = 0.76, CIs [0.74–0.77]). For the remaining two descriptors, no significant relationships emerged (“calm”: χ^2^(1) = 1.18, *p* = .278, OR = 1.02, CIs [0.99–1.04]; “posh”: χ^2^(1) < 0.001, *p* = .983, OR = 1.00, CIs [0.98–1.02], see Fig. 8a, bottom row).

#### 6.1.2 Faces

For faces, the GLMMs showed that there were significant negative relationships between descriptor frequency and 5 of the 6 descriptors. Specifically, there were significant negative relationships between descriptor frequencies and discrimination judgements of age (“young”: χ^2^(1) = 261.07, *p* < .001, OR = 0.81, CIs[0.79;0.84]), degree of boredom (“bored”: χ^2^(1) = 48.37, p < .001, OR = 0.93, CIs[0.91;0.95]), hair colour (“blonde”: χ^2^(1) = 264.41, p < .001, OR = 0.78, CIs [0.75;0.81]), ethnicity (“white”: χ^2^(1) = 57.43, p < .001, OR = 0.85, CIs [0.81;0.88]), and tiredness (“tired”: χ^2^(1) = 232.77, p < .001, OR = 0.85, CIs[0.83;0.87]). Only the model for the sex judgement data showed no significant relationship between descriptor frequency and discrimination judgements (“female”: χ^2^(1) = 0.15, *p* = .699, OR = 0.99, CIs[0.97;1.02], Fig. 8b, bottom row).

For descriptors for which there was a significant negative relationship, the results therefore show that the larger the difference in descriptor frequencies from Experiment 1 between the two voice/faces in a pair, the likelier it was that participants chose the voice/face that was described more frequently as e.g., “young” as indeed being the younger voice/faces in the discrimination task.

### 6.2 Can differences in descriptor position describe how identities differ from one another?

When investigating how descriptor position may relate to voice discrimination judgements, there were positive relationships in 3 of the 6 descriptors. As such, positive relationships were found for judgements of age (“young”: χ^2^(1) = 79.64, *p* < .001, OR = 1.65, CIs[1.47–1.84]), sex (“female”: χ^2^(1) = 32.04, p < .001, OR = 1.36, CIs[1.22–1.52]), degree of calmness (“calm”: χ^2^(1) = 51.53, p < .001, OR = 1.43, CIs [1.29–1.58]), and boredom (χ^2^(1) = 349.61, *p* < .001, OR = 0.76, CIs [0.74–0.77]). For the remaining 3 descriptors, no significant relationships emerged (educated: χ^2^(1) = 3.73, *p* = .053, OR = 1.10, CIs [1.00–1.20]; posh: χ^2^(1) = 3.53, *p* = .061, OR = 1.09, CIs[1.00–1.20]; “bored”: χ^2^(1) < 0.02, *p* = .885, OR = 1.01, CIs[0.90–1.12]; see Fig. 9a, bottom row). Although, relationships for “educated” and “posh” were not significant (p. = .061 and p = .053), these non-significant relationships observed in these models were positive in nature, thus showing trends in the expected directions.

#### 6.2.1 Faces

For faces, the GLMMs showed that there were significant positive relationships between descriptor position and face discrimination judgements for 4 of the descriptors. There were significant positive relationships for the judgements of the age (“young”: χ^2^(1) = 6.86, *p* = .009, OR = 1.15, CIs[1.03;1.27]), hair colour (“blonde”: χ^2^(1) = 7.62, *p*= .006, OR = 1.25, CIs[1.07;1.46]), ethnicity (“white”: χ^2^(1) = 105.78, *p* < .001, OR = 1.80, CIs[1.60;2.03]), and tiredness (χ^2^(1) = 7.19, *p*= .007, OR = 1.15, CIs[104;1.28]). There was no relationship between the descriptor position and discrimination judgements for degree of boredom (“bored”: χ^2^(1) = 1.29, *p* = .256, OR = 1.06, CIs[0.96;1.16]. Surprisingly, furthermore, the sex judgement data showed a significantly negative relationship between descriptor position and discrimination judgements (“female”: χ^2^(1) = 32.78, *p* < .001, OR = 0.76, CIs [0.69;0.83], see Fig. 9b bottom row).

These findings linking descriptor position to discrimination responses are in line with the findings for descriptor frequency. For descriptors for which a significant positive relationship was found, the models show that the larger the difference in descriptor position from Experiment 1 between the two voice/face in a pair, the likelier the participants were to choose the voice/face that was described earlier in time as e.g., “young” as being the younger voice/faces in the discrimination task.

### 6.3 Can we detect relationships between frequency and position measures and discrimination judgements across modalities?

#### 6.3.1 Descriptor frequency

The GLMMs showed that for 2 out of the three (“young”, “bored”), differences in descriptor frequencies could significantly predict participants’ responses, as indicated by the negative relationships plotted in Fig. 10 (top half, bottom row).

Specifically, there were negative relationships between descriptor frequencies and judgements of age (“young”: χ^2^(1) = 202.14, p < .001, OR = 0.47, CIs[0.41–0.52]) and boredom (“bored”: χ^2^(1) = 92.46, p <-.001, OR = 0.60, CIs[0.54–0.67]). For judgements of sex, no significant relationships emerged (“female”: χ^2^(1) = 0.20, *p*. = .652, OR = 1.02, CIs [0.93–1.11).

For the descriptors for which there were negative relationships in this analysis, these results therefore indicate that the larger the difference in descriptor proportion between the two identities, the more likely that participants identified the voice or face that received more instances of e.g., the descriptor “young”, as indeed being the younger voice or face in the discrimination task.

#### 6.3.2 Descriptor position

The GLMMs showed that for 2 out of the 3 descritpors (“young”, “female”), differences in descriptor position could significantly predict participants’ responses, as indicated by the positive relationships plotted in Fig. 10 (bottom half, bottom row).

Specifically, there were positive relationships between descriptor position and judgements of age (“young”: χ^2^(1) = 7.82, *p* = .005 OR = 1.14, CIs[1.04–1.24]) and sex (“female”: χ^2^(1) = 4.91, *p* = .027, OR = 1.10, CIs[1.01–1.20]). For judgements of boredom, there was – unexpectedly – a significantly negative relationship (“bored”: χ^2^(1) = 41.02 p < .001, OR = 0.74, CIs[0.68–0.81).

For the descriptors for which there were positive relationships in this analysis, larger the difference in descriptor position between the two identities result in participants being more likely to identify the voice or face for whom e.g., the descriptor “young” was mentioned earlier on in the list of descriptors, as indeed being the younger voice or face in the discrimination task.

Overall, these results indicate that free descriptor data (like explicit discrimination data) may be able to tap into higher-level, amodal (or indeed multimodal) representations of what it means that a person is for example, young, female or bored sounding/looking.

## 7 Discussion

Experiment 2 linked measures derived from free descriptors data to experimental discrimination responses and found significant relationships between these two types of data. The findings thus have several conceptual and methodological implications: 1) These findings show that free description data indeed encode information about how identities differ from one another. This could be interpreted as evidence for the two tasks tapping into similar kinds of representations of person characteristics. Finding relationships for crossmodal judgements may then further suggest that the tasks indeed tap into high-level representations that are not modality-specific. 2) Finding significant relationships between the measures derived from the free descriptors also supports the proposal from Experiment 1 that descriptor frequency and descriptor position may track how salient or prominent a characteristic is for a specific identity – and by extension for person perception in general. 3) By linking free description data to more commonly used discrimination data, the findings from Experiment 2 also help to contextualise free description data within the broader literature on person perception from voices and faces.

Although there were a similar number of significant relationships between discrimination judgements and the relative differences in descriptor frequency and descriptor salience, descriptor frequency appears to be an overall better predictor of voice and face discrimination responses than descriptor position, with relationships appearing to be overall stronger for models of descriptor frequency (see the reported odds ratios for these models as well as the steepness of slopes in Fig. 8 – 10). Interestingly, however, descriptor frequency and descriptor position appear to be partially complementary to one another: Although no significant relationship for voices emerged between descriptor frequency and discrimination judgements for the degree of calmness, there was a significant relationship when looking at this descriptor via descriptor position for voices.

**Fig. 4:**
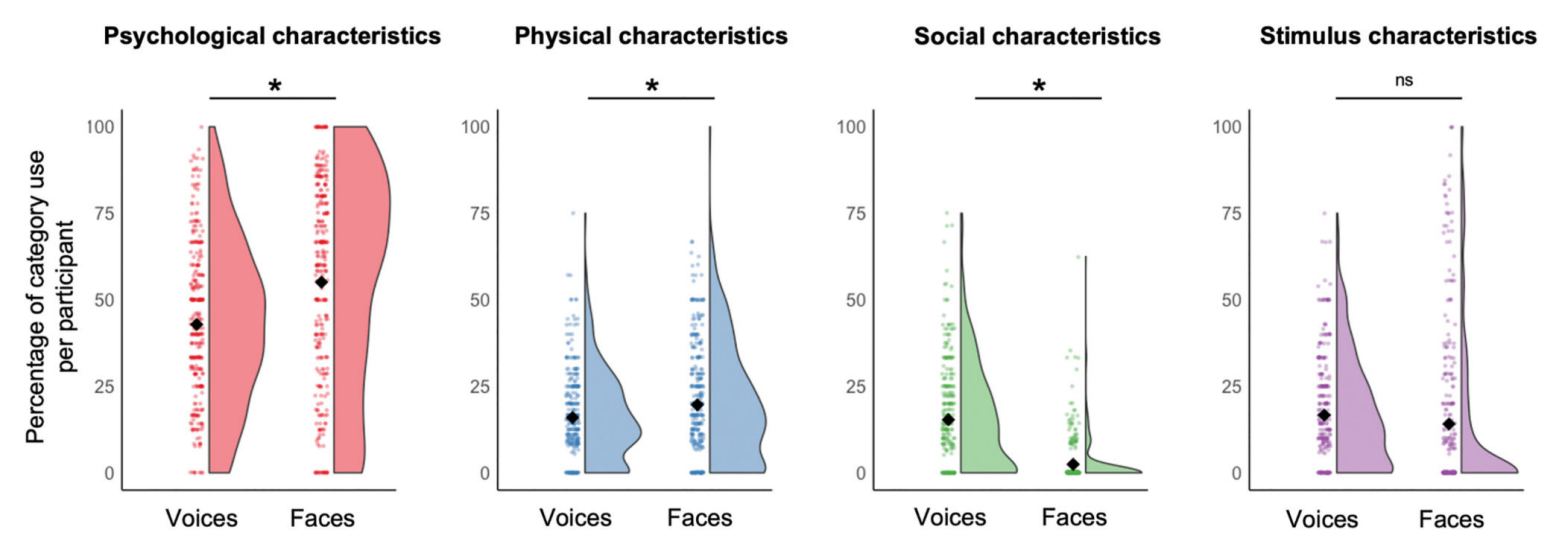
The percentage of descriptors used by participants for psychological characteristics, physical characteristics, social characteristics, and stimulus characteristics plotted for voices and faces respectively. Diamonds indicate the mean percentage across all participants, dots show the individual participant’s data to illustrate the spread of the underlying data alongside the half-violin plots.

**Fig. 5:**
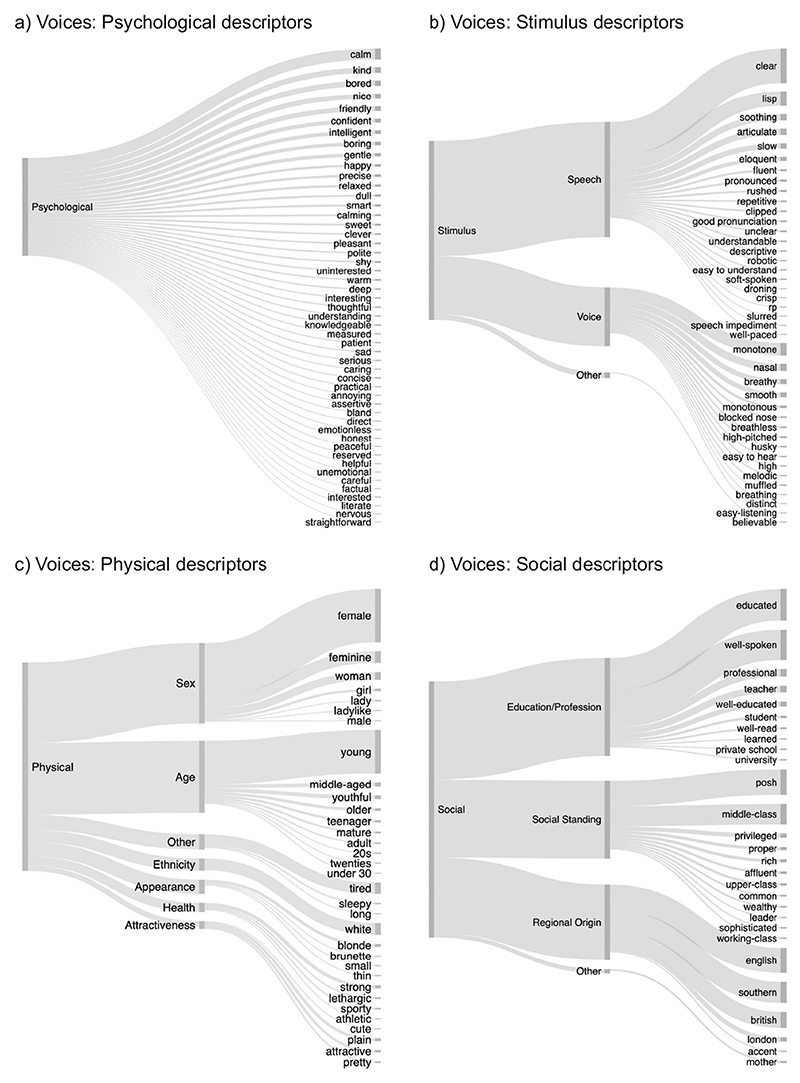
Sankey plots showing the classifications of a) psychological, b) stimulus, c) physical, and d) social descriptors for voices. The thickness of the connecting lines is proportional to the frequency with which different descriptors or characteristics were used. Sankey plots were created via sankeymatic.com.

While there were significant relationships for most of the voice and face descriptors examined, for some descriptors observed no relationship between descriptor frequency/position and discrimination data emerged. Additionally, two significant relationships in the opposite direction to the predictions emerging for sex judgements in relation to descriptor position in faces and crossmodal judgements. There are various possible explanations for this lack of expected relationships: For example, although “posh” for voices was the most frequently used descriptor in the category of social characteristics for voices, related terms such as “middle-class” or even “upper-class” were also listed at times. The same is true for “calm”, where “calming” and “relaxed” were also frequently chosen descriptors for voices (see also “bored” vs “boring”, Figs. 4 and 5). As such, relationships may have been diluted by not accounting for the presence of these related descriptors in the derived measures. This explanation, however, does not seem to readily explain the lack of a relationship observed for sex (i.e., the descriptor “female”) for faces, as fewer alternative descriptors of the person’s sex were available to participants. An alternative explanation for why no or only weak significant relationships were found for some descriptors may also lie in the derived measures of the difference in descriptor frequency and positions themselves: These measures are relatively crude in the sense that they only have a limited resolution and will therefore be likely noisy and/or imprecise and may not allow for an estimation of the true effect size. This issue could be resolved by having a much larger data set with more – and more varied – voice and face identities, each described by a larger number of participants. Increasing the number of stimuli and identities would result in more data points being available (e.g., more pairs of identities) while also increasing the variability and spread of difference measures. With this increased resolution of the measure, the power to detect any relationships to e.g., the discrimination data examined here should also increase. Nonetheless, finding numerous significant relationships between measures derived from free description data and an experimental discrimination task in the current study, despite the limited resolution of the measures derived from the free descriptions data, is a promising first step that shows that even substantially different types of data appear to at least tap into partially similar, higher-order representations and concepts. How much and what kind of representational information is shared among these types of data is unclear, such that further work is required.

## 8 General discussion

The current study set out to examine and characterise how humans describe other people based on their voices and faces, outlining similarities and differences between the two modalities. The broad implications of the findings from Experiments 1 and 2 are therefore two-fold: These experiments have on the one hand added to the knowledge of how voices and faces are described by taking a broad perspective on person perception, looking at more than one modality, and also (re-)investigating person perception largely in the absence of the constraints usually imposed on participants’ responses. Additionally, the current findings constitute a methodological advance, by conducting a quantitative analysis of free descriptions and showing that various properties of the free descriptor data encode person-related information that is shared with data acquired through more commonly-used discrimination tasks. Going beyond the close discussions of the findings from Experiments 1 and 2, I will speculate below on potential mechanisms underpinning the current results from the free description task, the potential for generalisability of the current findings beyond the small number of identities investigated here, and limitations and implications of the current findings.

### 8.1 Candidate processes and mechanisms supporting free descriptions of other people

One aspect of the free description of other people that this study has not probed empirically or speculated about yet is the question about which (cognitive) processes free descriptions most likely reflect. Do they primarily reflect perceptual processes, are the results of higher-level decision making, or do general task demands mostly shape descriptions? It could be proposed that the speed (and potentially the reliability) with which different types of characteristics can be perceived and identified from voices and faces could at least partially explain why in the current study physical and psychological characteristics are listed both frequently and/or early on. Previous studies of voice and face perception report, for example, that the sex of a person – a physical characteristic – can be perceived rapidly and with high accuracy from a voice or a face (Barragan-Jason, Lachat, & Barbeau, 2012; Bachorowski & Owren, 1999; see also for age perception from faces: Dobs et al., 2019). Similarly, character traits are perceived quickly (and reliably across perceivers) in voices and faces alike, although these perceived character traits do likely not reflect a person’s true character (McAleer, Todorov, & Belin, 2014; Mileva & Lavan, 2022; Todorov, Pakrashi, & Oosterhof, 2009). Conversely, assessing a social characteristic, such as the regional origin of a person from their voice will often evolve on a longer timescale because listeners ideally need to hear several speech sounds if not words to reliably detect and identify a regional or non-native accent. These examples of perceptual time courses seem to align with some of the findings in Experiment 1, where physical characteristics (mainly referring to the age and sex of a person) and psychological were listed frequently in first/early positions, while e.g., social characteristics were listed overall less frequently in these early positions.

While the speed and ease with which participants can perceive characteristics could thus shed light on why physical and/or psychological characteristics are listed frequently and/or early in Experiment 1, this kind of explanation does not adequately capture how participants are likely to complete a free description task. The process of listing of descriptors works on a much slower time scale (2 minutes in this study) than is the case for the studies exploring the time course of e.g., age, sex or personality trait perception (usually on a < 1 s timescale). As such in the current study, several seconds may pass between the participants perceiving a characteristic and then choosing to write it down. I, however, speculate that participants were thus more likely to have perceived a number of properties simultaneously or in close succession to one another but will have then *chosen* to list the “best descriptors” to their minds. Which descriptors get chosen will then, however, depend on for example what the participants consider important for the task at hand (e.g., characteristics that are considered too obvious, such as “human”, may not get listed) or how easy participants find to describe their subjective impression. Due to these proposed higher-level decision-making processes, it is therefore still unlikely that there is a direct perceptual link between how quickly and reliably a person characteristic can be perceived and how early and frequently it may be listed by participants. As indicated by the results of Experiment 2, however, the order and frequency of descriptors in turn still appear to reflect their salience/prominence for the identity in question to some degree. It is therefore worth considering that there may be an indirect link, where person characteristics that are easy (and quick) to perceive may be more likely to be chosen as the “best descriptors” by participants.

**Fig. 6:**
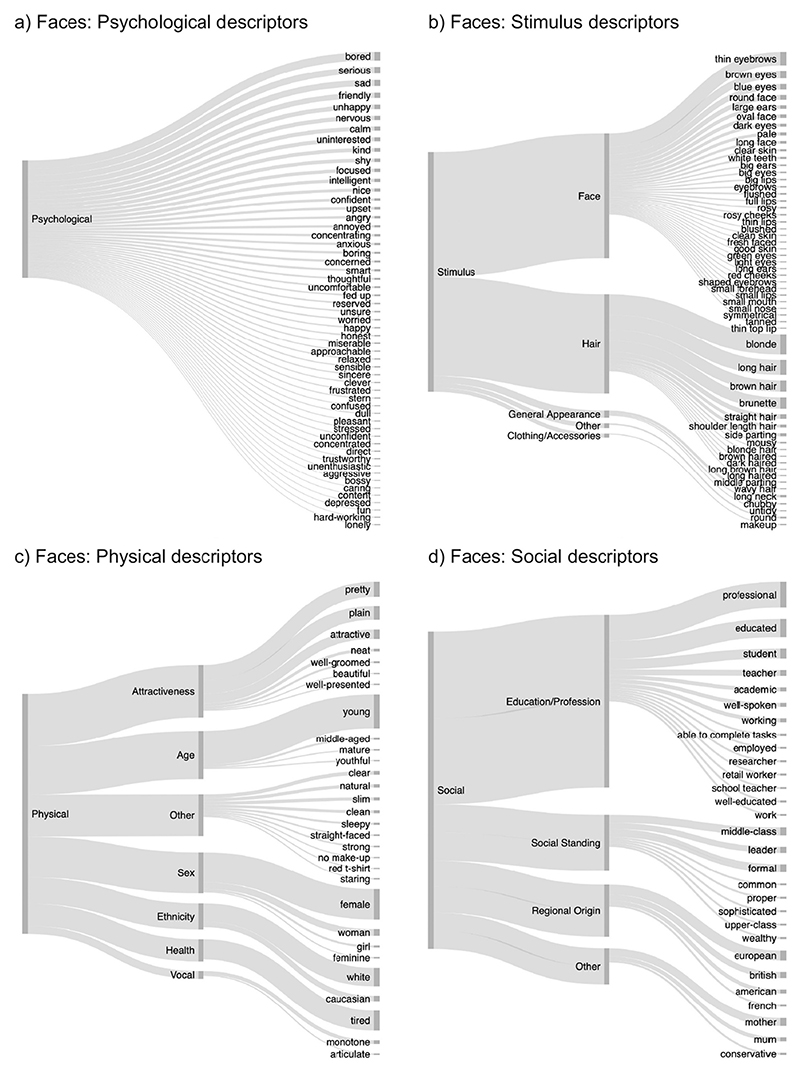
Sankey plots showing the classifications of a) psychological, b) stimulus, c) physical, and d) social descriptors for faces. The thickness of the connecting lines is proportional to the frequency with which different descriptors or characteristics were used. Sankey plots were created via sankeymatic.com.

**Fig. 7:**
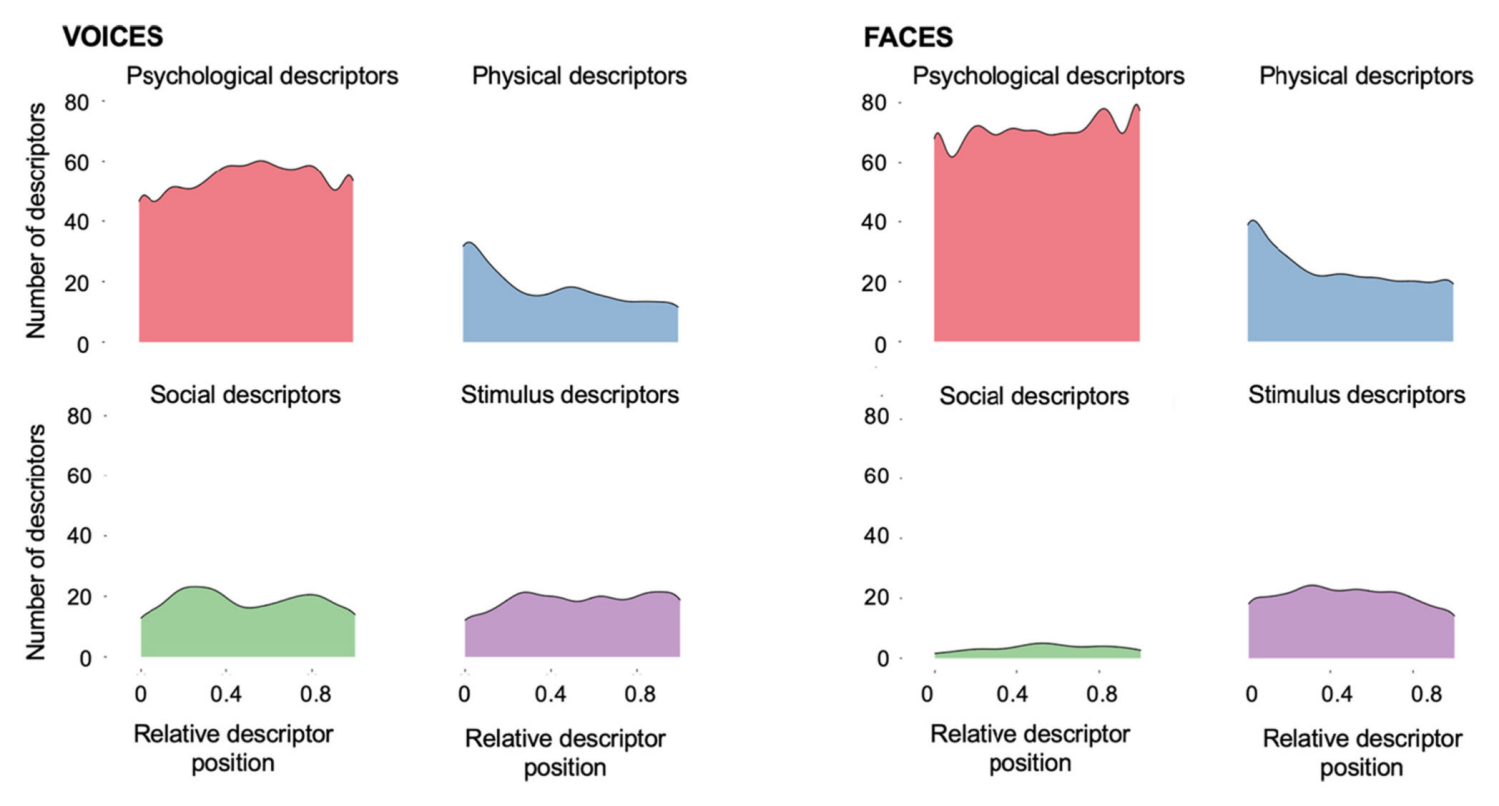
Density plots showing the frequency of descriptors per category plotted by their relative position, i.e., their relative time point of listing, for voices (lefthand side) and faces (righthand side).

**Fig. 8:**
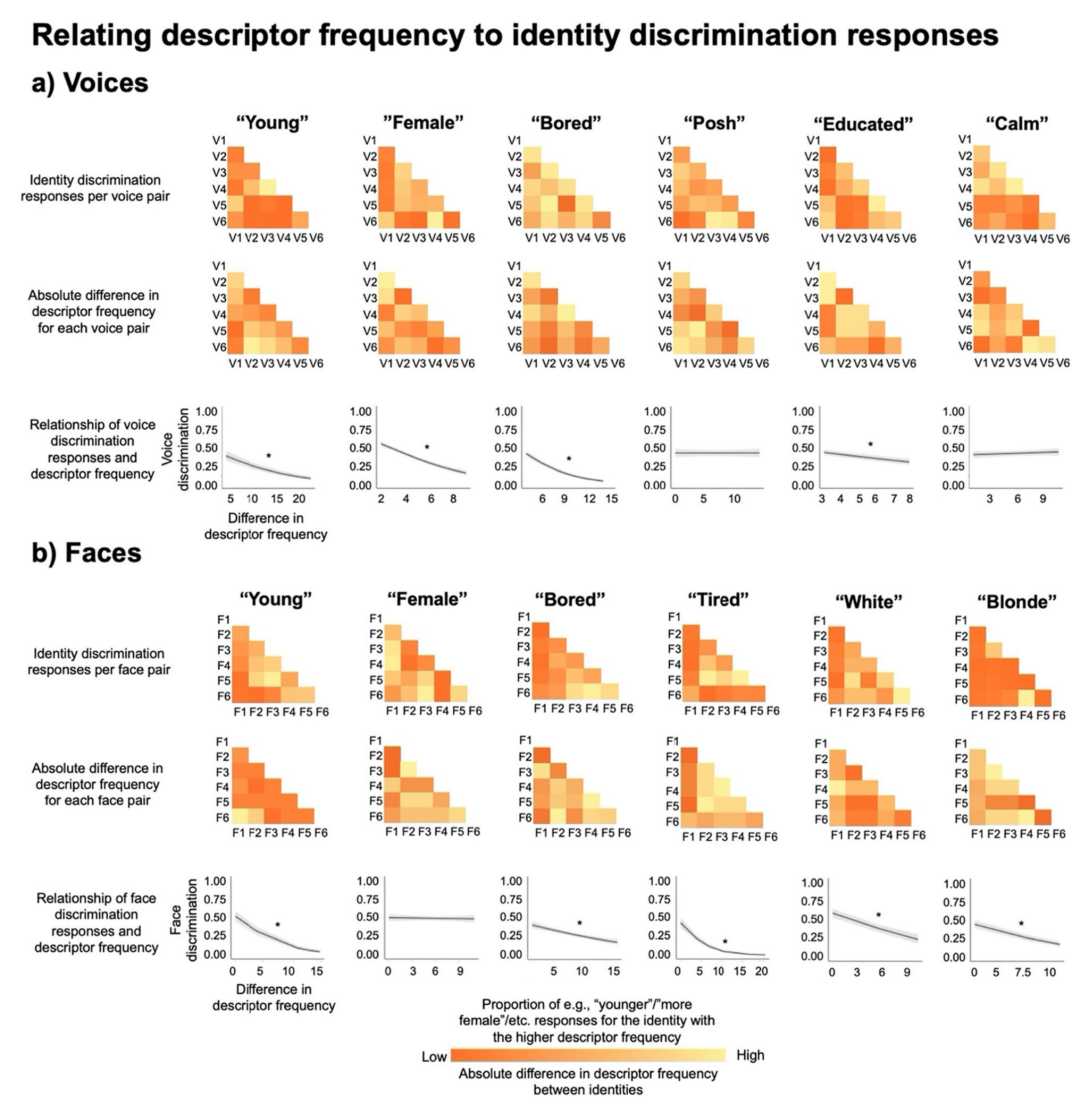
Matrices visualising the data for voices (panel a) and faces (panel b) from the discrimination tasks (top row) and the measure derived from the free descriptor task (absolute difference in descriptor frequency; middle row). The bottom row in both panels shows the relationship between discrimination responses and the relative descriptor frequency as predicted by the statistical models.

**Fig. 9:**
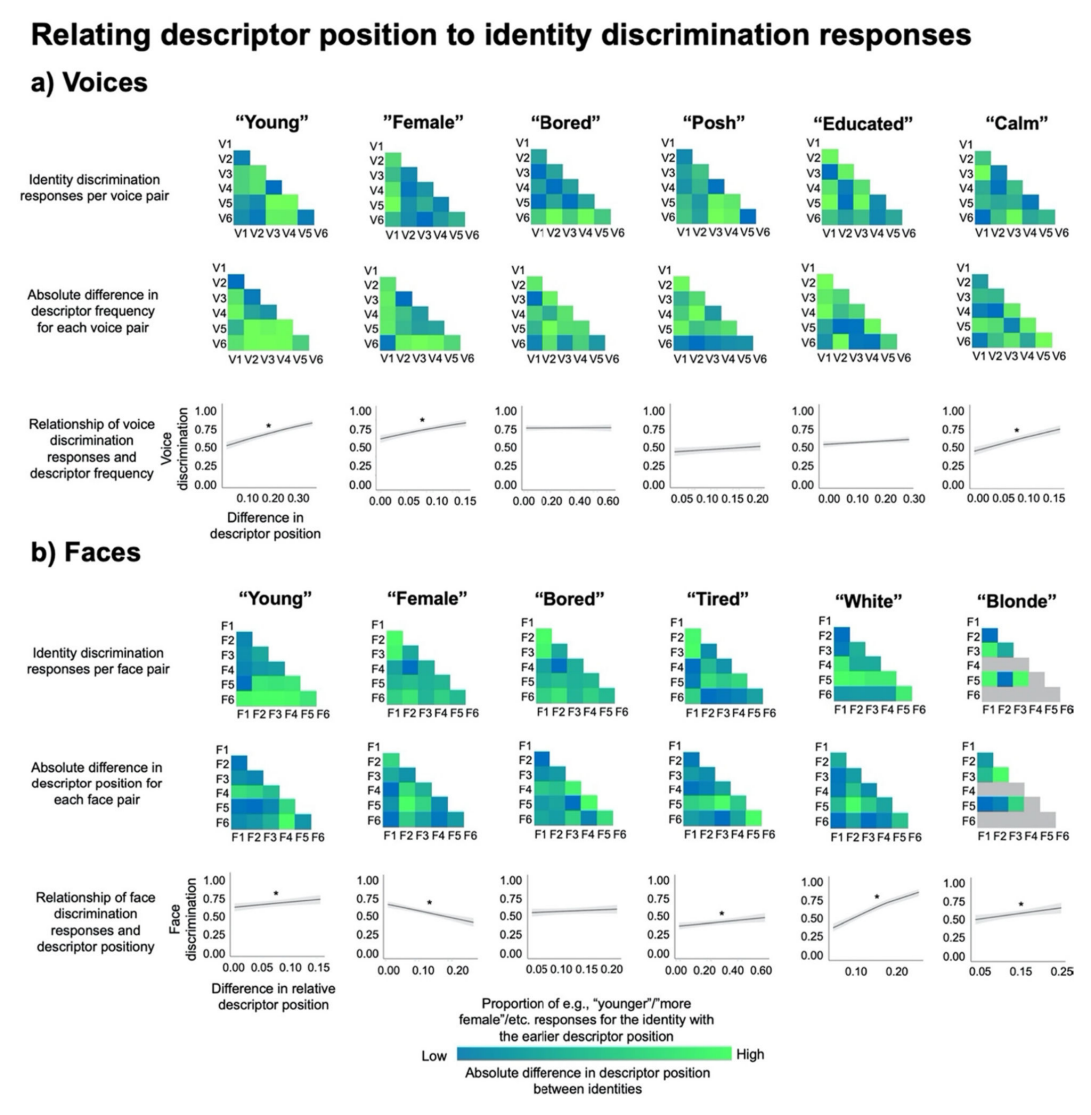
Matrices visualising the data for voices (panel a) and faces (panel b) from the discrimination tasks (top row) and the measure derived from the free descriptor task (absolute difference in descriptor position; middle row). The bottom row in both panels shows the relationship between discrimination responses and relative descriptor position as predicted by the statistical models.

**Fig. 10:**
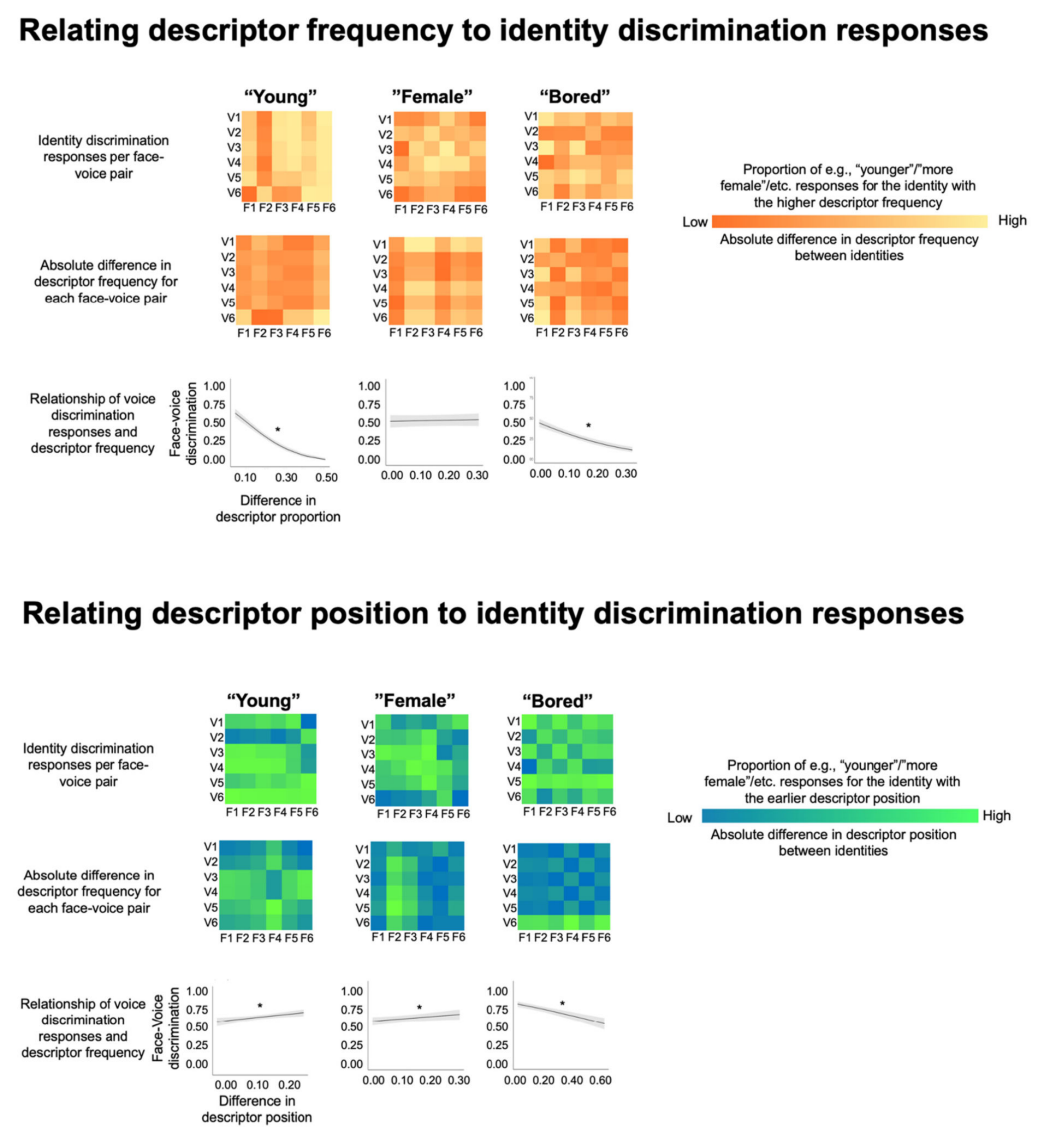
Matrices visualising the data for the crossmodal discrimination task (top row), and the measures derived from the free descriptor task (top half of the figure: absolute difference in descriptor frequency; middle row; bottom half of the figure: absolute difference in descriptor position; middle row).). The bottom row in both panels shows the relationship between discrimination responses and relative descriptor frequency or descriptor position as predicted by the statistical models.

An alternative explanation for these findings ties the observed data to inherent properties of free description data: There is, for example, only a relatively small number of commonly-used and suitable words describing a person’s sex (e.g., “female”, “woman”, “girl”, “feminine” from the current set of stimuli). Once one or two of these descriptors have been listed, participants will likely move on to describe other types of person characteristics (e.g., a person’s age or social characteristics). In contrast, for psychological character trait descriptions, many suitable and overlapping but not fully exchangeable words are available to describe people (e.g., “kind”, “friendly”, “affable”, “nice”), all of which participants may list. This explanation could then also account for why participants overall listed many more unique or rarely used descriptors falling under psychological characteristics than e.g., physical characteristics (see Figs. 4 and 5), why a consistently high number of psychological characteristics was listed throughout the 2-min presentation of the voice and face stimuli, and why most physical characteristics were mentioned early but not later in the participants’ descriptor lists (see Fig. 6).

Thus, the free descriptor data presented here likely reflect the outcome of a number of processes, where the data may reflect both cognitive processes associated with person perception but have been shaped by task demands and stimulus properties. It is worth noting that for example some of the similarities across modalities may be emergent from the nature of a free description task. At the same time, the many similarities in how voices and faces – and indeed different individual identities – are described, could, however, suggest that the findings of Experiment 1 may be generalisable beyond this specific experiment – at least at this coarse level of category-based analysis. If this is the case, the current findings may then suggest that humans use a common conceptual framework to impose a structure on their descriptions of other people. Similar proposals have previously been put forward by Fiske and Cox (1979) and Cantor and Mischel (1979), who suggest that (free) descriptions of other people may share commonalities with descriptions of objects in general. Furthermore, Osgood et al. (1957) suggest that humans structure the meaning of words based on weightings of a set of common dimensions, such as evaluations (positive vs negative), potency (strong vs weak), and activity (active vs inactive), which are also reflected in later work on low-dimensional face and voice trait spaces (e.g., McAleer et al., 2014; Oosterhof & Todorov, 2008). Intriguingly, for face trait spaces, it has been shown that (psychological) trait evaluations often follow a similar structure not only at group-level but also for individual perceivers (Sutherland, Rhodes, Burton, & Young, 2020, Sutherland, Wankel, & Hansel, 2020) and can be directly linked to the physical features of people’s faces (Vernon, Sutherland, Young, & Hartley, 2014). These similarities at the perceiver-level have been taken to reflect a ‘social reality’ that is to some degree shared across different perceivers that is derived from physical cues. While no detailed quantitative analysis of individual perceiver’s descriptions was conducted for the current data, it is nonetheless tempting to consider that similarities at group-level could indeed also reflect broad similarities across individual perceivers (while leaving room for individual differences, e.g., Germine et al., 2015; Hehman, Sutherland, Flake, & Slepian, 2017; Hönekopp, 2006; Sutherland, Rhodes, et al., 2020, Sutherland, Wankel, & Hansel, 2020). More work is, however, required to determine the underlying mechanisms for how we describe other people based on their voices and faces.

### 8.2 Generalisability of the current findings for free descriptions of other people

Our results based on the categorisation of descriptors appear to be relatively consistent across all the voices and faces examined, speaking to some degree of generalisability of findings. It is therefore interesting to consider how perceiver- or stimulus-specific characteristics could affect how people are described by others. This is particularly important because the current findings are based on only 6 identities per modality, with the voices and faces sampled in this experiment being drawn from a fairly homogenous set of identities. The identities sampled are therefore likely not representative of the broader population, which is a clear limitation of the current study. Indeed, the specific demographic properties of the sampled identities are reflected in the current data, most clearly in the individual descriptors: The identities sampled for voices were all young adults, female, with a Standard Southern British accent sampled from the university population (see Baker & Hazan, 2011). Thus, descriptors such as “young” or “posh” were used frequently for these voices (see Fig. 5). Similarly, the faces were all young, white females, sampled from the student population (Cooke et al., 2006), and as such “white” and “female” were used frequently. Across voices and faces, stimuli were neutral in emotional content, resulting in the “bored” being used very frequently (as opposed to e.g., “happy”/”friendly” which was the most frequent emotion-related descriptor in Lin, Keles, & Adolphs, 2021 and Sutherland et al., 2018). In a more heterogeneous set of stimuli (e.g., including individuals from across the full adult age range, sampling people from different genders, and/or including more socio-economic diversity), a different set of individual descriptors would have been used with different frequencies across voices and faces. Crucially, however, these proposed differences in descriptions introduced by stimulus sets with different properties is likely to be reflected in the *individual descriptors* and not necessarily in the types of properties described. As such, the overall distribution of how descriptors fall into the different categories may still show similar trends to the ones observed in the current data.

That is not to say that the types of descriptors mentioned will always resemble the pattern observed in the current study. Such changes in distribution could be brought about by interactions of stimulus and participant characteristics: For example, in Experiment 1, listeners describing the voices were all from the UK, listening to Standard Southern British English (SSBE) accents, which are often considered the modern-day equivalent of received pronunciation (RP; International Phonetics Association, 1999), making it a relatively standard accent in England. If the voices had featured a non-standard regional or non-native accent, the regional origin of the person would have potentially been more salient or distinguishable. It is therefore conceivable that the same sample of listeners may have described the perceived regional origin of non-standard-accented speakers more frequently than is the case in the current data, thus changing the distributions of descriptors across categories. How descriptors are used and chosen by the perceivers will therefore likely depend on what each perceiver considers to be salient within their frame of reference. Whether and how much such interactions of perceiver and stimulus characteristics affect the patterns of current results is unclear, such that additional work is required to answer these questions.

### 8.3 Conceptual implications and future work

The current study therefore demonstrates that there are strengths to using free description data to learn about how voices and faces are described. Perceivers are able to freely comment on whichever characteristics they deem to be salient. The resultant descriptions therefore provide a richer account of a person’s perceived characteristics than can be collected via more traditional experimental studies. Since Experiment 2 has shown that free description data can be linked to traditional tasks, the free descriptions could thus provide a fuller understanding of the properties of individual voices or faces. Aside from providing a platform for gleaning basic insights into person perception, such a rich understanding may be useful, for example, to select voices or faces with certain properties for experimental studies or commercial purposes. It is, however, also worth noting that while free description data may open up exciting new avenues for research, the data is complex and often not straightforward to analyse quantitatively, such that more methodological work is needed to establish how free description data can best be used for person perception research.

From a conceptual point of view, one of the most striking outcomes of the current study is the breadth of categories and subcategories of terms used to describe people from faces and voices. This underlines the intuitive notion that person perception is indeed likely a multivariate or multifaceted process: Participants spontaneously and without further instruction can perceive and describe multiple person characteristics upon seeing a face or hearing a voice. While the proposal that person perception is highly multivariate in its nature is not new (see Osgood et al., 1957; Holmgren, 1967; Vernon et al., 2014; Voiers, 1964), it is often not directly reflected in more recent experimental studies. For example, most experiments in experimental psychology study e.g., the perception of identity or traits or emotional expressions from faces or voices. Similar trends are apparent in the neuropsychological literature, where the focus of many studies is to determine whether a patient can recognise a familiar person, learn to recognise new identities, or discriminate between unfamiliar people based on their faces or voice (e. g., Gianotti, 2011 for a review; Roswandowitz, Kappes, Obrig, & von Kriegstein, 2018; Van Lancker & Kreiman, 1987; Van Lancker, Cummings, Kreiman, & Dobkin, 1988; Van Lancker, Kreiman, & Cummings, 1989). Although these kinds of targeted studies are a powerful tool to test specific hypotheses, they inevitably constrain and guide perceivers’ judgements to a specific aspect of person perception, thus neglecting the breadth and complexity of person perception. As a result, our understanding of e.g., phonagnosia, the inability to recognise familiar voices, is still largely focused on voice identity perception, while little is known about whether other aspects of person perception are also affected or remain intact (but see Garrido et al., 2009 or Le Grand et al., 2006 and Rezlescu, Susilo, Barton, & Duchaine, 2014 for prosopagnosia). I would therefore argue that the current study can be seen as a call to broaden the focus of future person perception research again.

Furthermore, the current findings put into focus the possibility that different person characteristics that are perceived either at the same time or in close succession may interact with another (e.g., Glaser & Düngelhoff, 1984), or that one percept may indeed help to trigger the next (see emotion overgeneralisation effects, e.g., Zebrowitz, 1996, 1997). Some studies have already described such interactions between physical characteristics, such as a person’s (perceived) age and sex, and psychological characteristics, such as perceived social traits, where for example dominance-related trait attributes are usually more clearly associated with male faces than with female faces (e.g., Oosterhof & Todorov, 2008). Similarly, co-dependencies between the perception of a person’s age and their attractiveness have been described, with younger faces being evaluated as more attractive on average than older faces (e. g., Kiiski, Cullen, Clavin, & Newell, 2016, Sutherland et al., 2013). For voices, correlations between some social traits (e.g., trustworthiness and attractiveness) but not between others (e.g., attractiveness and dominance) have also been described (Rezlescu et al., 2015). Furthermore, the current study finds that some types of descriptors and person characteristics appear to be more salient than others as indicated by being described earlier and/or more frequently. These findings broadly align with findings from trait evaluations studies, which report a relative primacy of warmth-related or trustworthiness-related trait characteristics over other traits (e.g., Brambilla, Rusconi, Sacchi, & Cherubini, 2011; Fiske, Cuddy, & Glick, 2007; Lin et al., 2021; McAleer, Todorov and Belin, 2014; Oosterhof & Todorov, 2008, but see Nauts et al., 2014).

These findings thus suggest that some person characteristics may be more likely than others to be perceived and described alongside one another for a voice or a face. Alternatively, it could be proposed that if one percept is more salient than another related property, the more salient characteristic might of course cancel also out the less salient characteristic in free descriptions tasks. How such associations and co-dependencies and interactions might manifest during naturalistic person perception is to date not yet well understood. More broadly speaking, however, these pieces of evidence suggest that not all characteristics are equal during person perception. Indeed, how people are perceived (and described) may be organised in a structured manner, where some characteristics are more immediate and/or important than others. It is to date unclear why or how such a structure may arise: A wide range of perceptual and cognitive processes may underpin some of this proposed structure. At the same time, cultural, societal, and situational factors may further shape person perception across different individuals (see Sutherland et al., 2015 for an example of how stereotypes can influence how people are evaluated). Which characteristics are relevant under which circumstances is a question that requires more attention to enable researchers to tie together existing findings about individual person characteristics and move towards a broad, unified account of person perception – from voices, faces, and beyond.

## Supplementary Material

Supplementary data

## Data Availability

The data for Experiment 1 and Experiemnt 2 are available on the OSF: https://osf.io/4wk7e/.
