# Supplementary material for: How do we describe other people from voices and faces?
Source: Cognition. Author manuscript; Available in PMC 2025 Sep 16. (PMC7618126; doi:10.1016/j.cognition.2022.105253)
Supplement: Supplementary data [file EMS208280-supplement-Supplementary_data.docx]

**Supplementary Analysis 1: Categorising free descriptors**

The descriptors collected for faces and voices in Experiment 1 were categorised by 384 lay judges (mean age: 40.6 years, SD = 12.9 years, 293 female) into four broad categories: Physical, Psychological, Social, and Stimulus characteristics. Lay judges were recruited from Prolific, were native speakers of English, who were born and were currently resident in the UK. These lay judges were therefore sampled according to the same criteria as the participants who provided the free descriptions of the faces and voices. Participants were reimbursed for their time.

Each lay judge was presented with an information sheet and consent form. After completing the consent form, lay judges were told that they will categorise a set of words that were collected as part of a study during which participants described people in their own words based on short recordings of their voices or videos of their faces. Lay judges were then asked to categorise a subset of the unique descriptors for voices or faces in relation to one of the categories (psychological, physical, social, or stimulus characteristics). Category assignment was random. A description of the category a lay judge was asked to use was presented to lay judges, with descriptions being based on the subcategories included in each category. For example, physical characteristics would be described as including “all words that describe a person’s age, sex, race, health, and all other aspects associated with their physical body” and contrasted with a selection of the subcategories from the other categories: “Physical characteristics do, however, not include words that describe a person’s emotions, moods, their level of education or social standing”. For their assigned category, lay judges then provided a binary yes/no response to express whether they thought a given word would fit that category or not (“Does this word describe someone’s [psychological characteristics/physical characteristics/social characteristics/[face/voice]?”).

Each lay judge classified around 180 descriptors, with each individual descriptor being thus classified by around 10 lay judges for each of the categories. From these ratings, the proportion of “Yes” responses for each category and each descriptor was computed. Each descriptor was therefore associated with a proportion value for each of the four categories. For example, the proportion of “Yes” responses for the descriptor “polite” for faces was 0.8 for psychological characteristics, 0.09 for physical characteristics, 0.33 for social characteristics, and 0 for a description of a person’s face. The proportion of “Yes” responses for “female” was 0.2 for psychological characteristics, 1.0 for physical characteristics, 0.17 for social characteristics, and 0.2 for a description of a person’s face. Descriptors with a proportion for “Yes” responses above 0.5 for one (and only one) of the categories were assigned to that particular category (e.g., from the example above, “polite” would be assigned to the category “Psychological characteristics). Descriptors with a proportion for “Yes” responses below 0.5 for all categories were assigned to “Other”.

For descriptors where two (or rarely more) categories had a proportion of > 0.5 for “Yes” responses (256 individual descriptors for voices and 214 for faces), categorisations of two expert judges (the author and another expert in voice identity perception) were used to determine the final category for these words: Two expert judges had previously independently classified the voice and face descriptors into categories without having been aware of the lay judge’s categorisations. For all but 14 voice descriptors and 6 face descriptors, expert judges had picked one of the categories that had been identified as being suitable from the lay judge’s categorisations (i.e., one of the categories receiving > 0.5 “Yes” responses). For the 14 voice and 6 faces descriptors where expert categorisations diverged from the categories identified by lay judges, expert judges revisited their categorisations and agreed on a final category.

In addition to the ambiguous descriptors that fit well within multiple categories, some descriptors were listed for both faces and voices. Since face and voice descriptors were rated independently by the lay judges, we therefore finally checked whether any descriptors that were used for both faces and voices were assigned to different categories (e.g., “fair” is more likely to describe someone’s fair or skin colour in the context of faces than for voices, where a psychological meaning might dominate for “fair”). This was the case for 22 unique descriptors. Similarly, for a further 33 descriptors, one category may have reached a proportion of > 0.5 “Yes” responses in one modality but did not reach this threshold in the other modality (thus being labelled as “Other”). In these cases, categorisations from the two expert judges were again used to classify the descriptors as one of the candidate categories based on the lay judges’ categorisations or, alternatively, labelling them as “Other” across both modalities. As a result of this process, the descriptors that are used for voices and faces alike are always associated with the same category for both modalities.

**Classifying free descriptors into subcategories**

The two expert judges furthermore classified all descriptors within their respective categories into sub-categories. Thus, descriptors labelled as referring to e.g., “Physical characteristics” were subcategorised as referring to a person’s age, sex, health, etc. (see Figure 1 in the main manuscript). Descriptors categorised as “Other” were not further subcategorised. After subcategorising all descriptors, judges compared their categorisations and resolved any disagreements.

While we initially attempted to classify psychological characteristics further into subcategories, in terms of emotions/moods, and personality traits (positive/neutral/negative). However, it became quickly apparent that these kinds of categorisations were largely subjective and we therefore refrained from subcategorising psychological characteristics further.

**Supplementary Analysis 2: Hierarchical clustering of the descriptors**

To provide an alternative, computational clustering approach to the human judgement-based approach that is presented in the main text, we applied a hierarchical agglomerative clustering (HAC) algorithm to our descriptor data in MATLAB, for faces and voices separately. For this purpose, we extracted vectors representing the meanings of each of our individual descriptors from the fastText library (Bojanowski et al., 2017). To exclude descriptors that are not representative of how voices and faces are described and may thus add noise to the clustering outcome, we only included descriptors that were mentioned more than 3 times (i.e., by around 1% of perceivers) in our voice and face description data respectively. Descriptors that were not part of the fastText library also were also excluded from this clustering analysis. This process resulted in unique 191 descriptors being considered for voices and unique 135 descriptors being considered for faces. We then used the linkage function in MATLAB to cluster the word vectors based on their average cosine similarity. This approach should therefore cluster any descriptors that are similar in their meaning (i.e., similar in their vectors) together. No cut-offs or thresholds were applied. Clustering solutions are visualised via dendrograms below for voices (Supplementary Figure 1) and faces (Supplementary Figure 2) respectively. The lower the number of the y axis for a common link in the dendrogram, the more similar descriptors are to one another.

The clustering solutions show that the HAC algorithm is indeed often able to identify words that are closely related (e.g., “calm”, “quiet”, and “peaceful” are clustered closely together). However, clusters become harder to interpret when trying to glean more general clusters. That is, while e.g., trait/psychological characteristics are largely combined in the same broadly defined cluster, the other categories appear to be either mixed together or broken up into very small clusters. Similarly, groupings of individual descriptors do not necessarily align for faces and voices, which is difficult given that the current study aims at directly comparing descriptions of faces and voices. It may have been possible to use the computational clustering solution as e.g., a basis for the categorisation with expert judges disambiguating category assignments. For the current study, a human-based categorisation approach was deemed more appropriate.


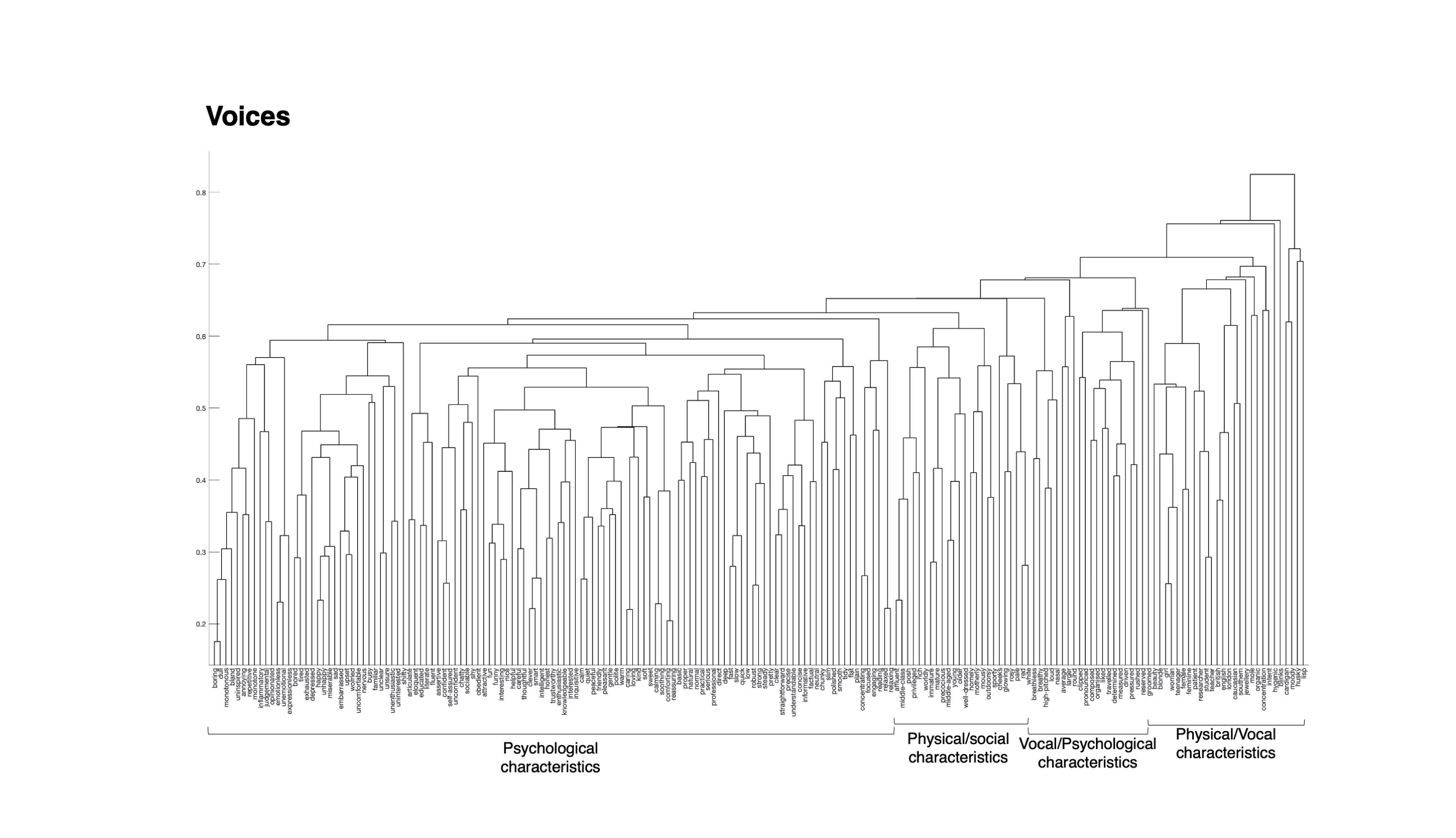


*Supplementary Figure 1: Dendrogram showing the clustering solution for 135 of the most frequent descriptors for voices. Each*

*descriptor was mentioned at least 3 times.*


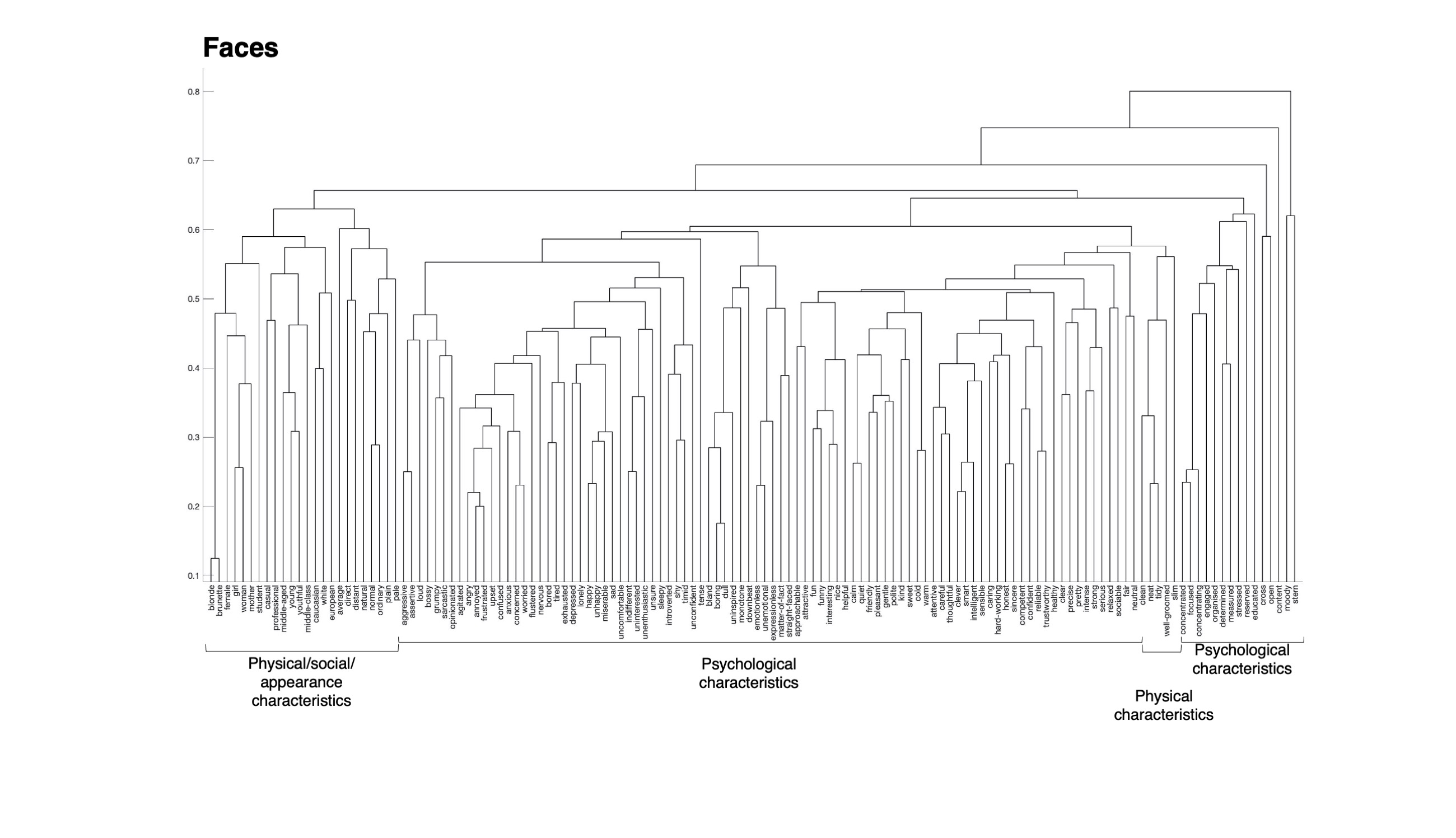


*Supplementary Figure 2: Dendrogram showing the clustering solution for 191 of the most frequent descriptors of faces. Each descriptor was mentioned at least 3 times.*

**Supplementary Analysis 3: Distributions of descriptors across the categories by identity**

Supplementary Table 1 Percentages for descriptors distributions across the different categories across all voice identities as well as for the 6 individual voice identities.

| **VOICES** | **All** | **V1** | **V2** | **V3** | **V4** | **V5** | **V6** |
| --- | --- | --- | --- | --- | --- | --- | --- |
| **Psychological** | 44.34% | 46.65% | 42.96% | 38.90% | 38.22% | 48.58% | 49.69% |
| **Stimulus** | 15.53% | 12.13% | 20.32% | 19.06% | 17.43% | 15.03% | 9.98% |
| **Physical** | 15.53% | 15.48% | 12.93% | 15.93% | 16.44% | 17.43% | 14.87% |
| **Social** | 15.13% | 18.41% | 12.01% | 15.67% | 18.61% | 11.55% | 13.85% |
| **Other** | 9.46% | 7.32% | 11.55% | 9.66% | 9.31% | 7.41% | 11.61% |
|  |  |  |  |  |  |  |  |
|  |  |  |  |  |  |  |  |
| **FACES** | **All** | **F1** | **F2** | **F3** | **F4** | **F5** | **F6** |
| **Psychological** | 53.64% | 65.43% | 65.43% | 47.48% | 46.79% | 55.60% | 50.21% |
| **Physical** | 19.35% | 15.82% | 15.82% | 21.13% | 24.10% | 17.43% | 17.93% |
| **Stimulus** | 15.75% | 10.16% | 10.16% | 19.72% | 15.26% | 16.60% | 20.89% |
| **Other** | 8.60% | 5.47% | 5.47% | 9.46% | 8.23% | 9.34% | 8.44% |
| **Social** | 2.65% | 3.13% | 3.13% | 2.21% | 5.62% | 1.04% | 2.53% |

**Supplementary Analysis 4: Analysis of the distribution of descriptors across the different subcategories**

*Supplementary Table 2 Percentages for descriptors distributions across the different subcategories across all identities as well as for the six individual voice identities.*

| **VOICES** |  | **All** | **V1** | **V2** | **V3** | **V4** | **V5** | **V6** |
| --- | --- | --- | --- | --- | --- | --- | --- | --- |
| **Physical** | **Sex** | 38.41% | 37.84% | 41.07% | 39.34% | 39.76% | 35.00% | 38.36% |
|  | **Age** | 34.66% | 37.84% | 23.21% | 32.79% | 25.30% | 38.75% | 47.95% |
|  | **Ethnicity** | 5.85% | 4.05% | 5.36% | 3.28% | 8.43% | 2.50% | 5.48% |
|  | **Appearance** | 5.62% | 6.76% | 5.36% | 11.48% | 4.82% | 6.25% | 2.74% |
|  | **Health** | 4.45% | 0.00% | 7.14% | 3.28% | 4.82% | 11.25% | 0.00% |
|  | **Attractiveness** | 3.98% | 8.11% | 3.57% | 3.28% | 2.41% | 2.50% | 4.11% |
| **Stimulus** | **Speech** | 63.70% | 77.59% | 53.41% | 76.71% | 60.23% | 56.52% | 65.31% |
|  | **Voice** | 32.79% | 22.41% | 45.45% | 23.29% | 38.64% | 30.43% | 30.61% |
| **Social** | **Education/Profession** | 38.22% | 34.62% | 34.62% | 30.85% | 45.28% | 36.76% | 36.76% |
|  | **Social standing** | 30.53% | 23.08% | 23.08% | 40.43% | 15.09% | 26.47% | 26.47% |
|  | **Regional Origin** | 29.33% | 36.54% | 36.54% | 26.60% | 37.74% | 35.29% | 35.29% |

For voices, physical descriptors most often referred to the sex (38.4% of physical descriptors) and age of a person (34.7%), followed by descriptions of the ethnicity (5.9%), appearance (5.6%), health status (4.5%) and attractiveness (4.0%, see Figure 2c in the main manuscript). Stimulus descriptors included most often referred to as speech characteristics (63.7% of stimulus descriptors), followed by voice quality descriptions (32.8%; see Figure 2b in the main manuscript). Finally, social descriptors were split relatively evenly into terms referring to the education or profession of a person (38.2%), social standing (30.5% of social descriptors), and regional origin (29.3%, see Figure 2d in the main manuscript).

Similar distributions of descriptors among the subcategories are also apparent across most of the 6 voices tested, with some minor deviations. For example, while for Voice 3 40.4% of all social descriptors referred to social standing, social standing made up only 15.1% of social descriptors for Voice 4. We note, however, that there is only a relatively small number of descriptors in some of the subcategories, such that similarities and differences at this level need to be interpreted with some caution. Percentages of descriptors per voice for the subcategories are provided in Supplementary Table 2.

Supplementary Table 3 Percentages for descriptors distributions across the different subcategories across all face identities as well as for the six individual face identities.

| **FACES** |  | **All** | **F1** | **F2** | **F3** | **F4** | **F5** | **F6** |  |
| --- | --- | --- | --- | --- | --- | --- | --- | --- | --- |
| **Physical** | **Attractiveness** | 21.88% | 12.87% | 25.93% | 32.38% | 25.00% | 21.43% | 11.76% |  |
|  | **Age** | 20.14% | 21.78% | 22.22% | 20.00% | 17.50% | 22.62% | 17.65% |  |
|  | **Sex** | 17.19% | 21.78% | 12.35% | 13.33% | 19.17% | 19.05% | 16.47% |  |
|  | **Ethnicity** | 10.94% | 3.96% | 11.11% | 9.52% | 15.00% | 11.90% | 14.12% |  |
|  | **Health** | 9.20% | 20.79% | 2.47% | 5.71% | 5.83% | 3.57% | 16.47% |  |
|  | **Vocal** | 3.30% | 3.96% | 2.47% | 3.81% | 4.17% | 3.57% | 1.18% |  |
| **Stimulus** | **Face** | 52.03% | 54.69% | 53.85% | 60.20% | 47.37% | 38.75% | 55.56% |  |
|  | **Hair** | 41.36% | 34.38% | 44.23% | 36.73% | 44.74% | 53.75% | 36.36% |  |
|  | **General Appearance** | 2.99% | 3.13% | 1.92% | 0.00% | 3.95% | 3.75% | 5.05% |  |
|  | **Clothing/Accessories** | 1.71% | 3.13% | 0.00% | 3.06% | 0.00% | 1.25% | 2.02% |  |
| **Social** | **Education/Profession** | 54.43% | 28.57% | 56.25% | 63.64% | 57.14% | 40.00% | 58.33% |  |
|  | **Social standing** | 17.72% | 28.57% | 31.25% | 0.00% | 14.29% | 20.00% | 16.67% |  |
|  | **Regional Origin** | 16.46% | 28.57% | 6.25% | 36.36% | 17.86% | 20.00% | 0.00% |  |

For faces, psychological descriptors also included a wide range of character traits, social traits, and emotional and mood states (see Figure 3a in the main manuscript). 21.9% of descriptors referred to descriptions of the person’s attractiveness. Physical descriptors often referred to the age (20.1% of physical descriptors) and sex of a person (17.2%). Aside from these three types of physical characteristics, participants listed descriptors in relation to a person’s health status (10.9%), ethnicity (9.2%) and – despite the videos having been muted – vocal characteristics of a person (3.3%, see Figure 3c in the main manuscript). Stimulus descriptions included a range of descriptions of facial features (52.0% of descriptors of stimulus), followed by descriptions of a person’s hair (41.4%). 3.0% of descriptors of an appearance referred to the general appearance of a person (e.g., “long neck”) and 1.7% of descriptors referred to a person’s clothing or accessories (see Figure 3b in the main manuscript). Finally, social descriptors were most frequently referred to the education or profession (54.5% of social descriptors). This was followed by descriptions of social standing (17.7%) and regional origin (also 16.5%). Percentages of descriptors per face for the subcategories are provided below in Supplementary Table 1b.

*Voices vs faces*

Due to the relatively low number of data points in some of the subcategories, no statistical analyses were conducted to compare how faces and voices were described at the level of subcategories. Descriptively speaking, participants comment on the age and sex of a person relatively frequently for voices and faces alike. However, participants appear to comment more frequently on the age and sex of a person when describing physical characteristics for voices than for faces (age: 34.5% of physical descriptors for voices vs 20.1% for faces; sex: 38.8% of physical descriptors for voices vs 17.2% for faces). Strikingly, descriptors related to a person’s attractiveness (e.g., “beautiful”, “pretty”, “sexy”) made up 21.9% person of physical descriptors for faces and only 2.8% of physical descriptors for voices. Similarly, in the context of overall fewer descriptions of social characteristics in general, participants comment relatively more frequently on social standing, listing social class or standing attributes such as “posh” or “middle-class” for voices, while similar descriptors were less frequent for faces (voices: 30.4% vs faces: 17.2%).

**Supplementary Analysis 5: Ranges of the measures derived from the free description data (difference in frequency and difference in position) for Experiment 2**

*Supplementary Table 4 Overview of the ranges for the derived measures (difference in frequency and difference in position) for Experiment 2*

| **Voices** | **Frequency** | | **Position** | |
| --- | --- | --- | --- | --- |
|  | **Min** | **Max** | **Min** | **Max** |
| **Young** | 2 | 27 | 0.00 | 0.35 |
| **Female** | 1 | 9 | 0.00 | 0.17 |
| **Bored** | 1 | 14 | 0.00 | 0.74 |
| **Posh** | 0 | 14 | 0.01 | 0.21 |
| **Educated** | 0 | 8 | 0.01 | 0.31 |
| **Calm** | 1 | 11 | 0.00 | 0.17 |
|  |  |  |  |  |
| **Faces** | **Frequency** | | **Position** | |
|  | **Min** | **Max** | **Min** | **Max** |
| **Young** | 1 | 17 | 0.01 | 0.21 |
| **Female** | 0 | 11 | 0.01 | 0.27 |
| **Bored** | 1 | 17 | 0.01 | 0.21 |
| **Tired** | 0 | 19 | 0.01 | 0.57 |
| **White** | 0 | 9 | 0.01 | 0.28 |
| **Blonde** | 0 | 21 | 0.04 | 0.25 |
| **Crossmodal** | **Frequency** | | **Position** | |
|  | **Min** | **Max** | **Min** | **Max** |
| **Young** | 1 | 17 | 0.01 | 0.21 |
| **Female** | 0 | 11 | 0.01 | 0.27 |
| **Bored** | 1 | 17 | 0.01 | 0.21 |
